# A new quantum approach to binary classification

**DOI:** 10.1371/journal.pone.0216224

**Published:** 2019-05-09

**Authors:** Giuseppe Sergioli, Roberto Giuntini, Hector Freytes

**Affiliations:** 1 University of Cagliari, Cagliari, Italy; 2 Centro Linceo Interdisciplinare “B. Segre”, Roma, Italy; University of Queensland, AUSTRALIA

## Abstract

This paper proposes a new quantum-like method for the binary classification applied to classical datasets. Inspired by the quantum Helstrom measurement, this innovative approach has enabled us to define a new classifier, called *Helstrom Quantum Centroid* (HQC). This binary classifier (inspired by the concept of distinguishability between quantum states) acts on density matrices—called *density patterns*—that are the quantum encoding of classical patterns of a dataset. In this paper we compare the performance of HQC with respect to twelve standard (linear and non-linear) classifiers over fourteen different datasets. The experimental results show that HQC outperforms the other classifiers when compared to the Balanced Accuracy and other statistical measures. Finally, we show that the performance of our classifier is positively correlated to the increase in the number of “quantum copies” of a pattern and the resulting tensor product thereof.

## 1 Introduction

In the past few decades, various methods based on quantum information theory have been used extensively to focus on a variety of problems concerning classification and clustering [1–4]. On the other hand, some classification methods developed in computer engineering have been employed to solve such problems as quantum-state discrimination [5–8], which are closely connected with certain recent developments in quantum cryptography. In view of these exchanges, quantum computation and machine learning are nowadays recognized as two closely-related connected research fields. A natural starting point for bridging these two different topics is to establish a common background. The initial idea was to represent classical patterns in terms of quantum objects, with an eye to increasing the computational efficiency of the classification algorithms. Following this intuition, in the past few years many attempts have been made to apply the quantum formalism to signal processing [9] and pattern recognition [10, 11].

A recourse to quantum states to represent classical patterns is naturally motivated by the possibility of exploiting the potential of quantum algorithms to boost the classification process. By way of example, it has been demonstrated that quantum algorithms can be used to reduce the time complexity of the standard k-nearest neighbor (kNN) classifier. Using the algorithms introduced in [3] (i.e., a quantum computational counterpart of the kNN classifier), it is possible to yield a polynomial reduction in query complexity compared to the corresponding classical algorithm. Extensive surveys concerning the applications of quantum computing in computational intelligence and machine learning can be found in [4, 12]. It is thought these approaches may lead to computational advantages from a quantum perspective [13, 14].

A different line of research, however, consists in using quantum formalism to obtain significant benefits in the classical computational contexts. We intend to explore this idea and provide a model aimed at processing binary classical datasets (in a supervised system), i.e., dataset containing only two classes of different objects (it should be remembered that there are several methods [15] to treat multiple class classification as a suitable combination of binary classifications).

The architecture of our model comprises the following three steps: *i*) *encoding*: each element (object, observation) of the dataset is encoded into a density operator, which is the standard mathematical tool to formally describe a quantum state; *ii*) *classification*: i.e., the application of a “quantum-inspired” version of a standard binary classifier on the encoded dataset is applied; *iii*) the result of the classification process is decoded in the initial classical space. The experimental setting is based on the application of our models to artificial and real datasets available from standard machine learning repositories.

A number of recent works [16–18] have introduced a “quantum-inspired” classifier—named *Quantum Nearest Mean Classifier* (QNMC)—based on the Nearest Mean Classifier (NMC). These works have highlighted the benefits of the QNMC on both artificial and real datasets, and in a biomedical context, in particular, QNMC has been employed to detect the pulmonary fibrosis in a dataset of suspected patients [19].

Here, we introduce a new binary classifier based on the Helstrom measurement [7] and provide a full comparison between this classifier and some other commonly used (linear and non linear) classifiers. The comparison is made by analyzing significant statistical quantities obtained by applying each algorithm to fourteen different datasets. We show that the new algorithm, on average, performs better than all the other competitors considered.

The paper is organized as follows: in Section 3 we describe the standard procedure to introduce a quantum-inspired classifier. In Section 4, we provide details of the so called Helstrom Quantum Centroid (HQC). Section 5 compares the performances of HCQ with other standard and commonly used classifiers by applying each of these on fourteen different datasets. Final remarks and further developments conclude the paper. All statistical data are listed in the Tables A-O in S1 File.

## 2 Quantum-inspired methods in the classification process

The general purpose of a classification process is to classify a set of objects, i.e., to assign to each object from the set, a label that represents real classes (for instance the class of the cats, the class of the cancer cells, the class of the human faces, etc). Following a standard classification procedure of supervised learning systems (i.e., learning from a training dataset of correctly labeled objects) we initially select the *d* features that characterize all the objects of a given dataset. Thus, each object is represented by a *d*-dimensional real vector $X=(x^{1},\ldots ,x^{d})\in R^{d}.$ Formally, we say that a pattern is represented by a pair (*X*_*i*_, *l*_*i*_), where *X*_*i*_ is the *d*-dimensional vector associated with the object and *l*_*i*_ labels the class of which the objects is a member. We consider a class as being merely a set of objects and we confine ourselves to the very common case where each object belongs to one class of objects only. Let *L* = {*l*_1_, …, *l*_*M*_} be the set of labels corresponding to their respective classes. The goal of the classification process is to design a *classifier* that will attributes (in the most accurate way possible) a label (a class) to any unlabeled object. The strategy is divided into two stages; first, training the classifier and second, performing the test proper. The dataset is therefore also divided into two parts: the first is used to train the classifier and the second is used to properly verify the accuracy of the classifier during a test [15, 20].

The aim of this work is to provide an original quantum-inspired version of the classical classification process and to show the extent to which this new model improves the accuracy (and also other significant statistical quantities) of the same process.

In order to provide a quantum approach to a standard classifier, the following three steps must be followed:

1. *Encoding*: a theoretical quantum object (i.e., a density operator in our case) is associated to each pattern, which we will call a *density pattern*;
2. *Classification*: we provide a quantum-inspired counterpart of standard classification procedure applied on a dataset of density operators (density patterns) instead of on a dataset of real vectors;
3. *Decoding*: we decode the results of the classification process in the domain of real vectors.

### 2.1 Encoding

It is well known that there are an infinite number of ways to encode a real vector into a density operator and the current state of the art suggests potential computational advantages of this sort. However, the relative advantages of each option may be strictly dataset-dependent: finding the “best” mode of encoding from real vectors to quantum states is still an open and complex problem. This is by no means surprising, given that (in accordance with the well known *No Free Lunch* Theorem [15]), in general no given classification method is superior in all aspects to all its competitors, because each dataset has its own unique and specific characteristics. Some recent works [16–18] have suggested different ways to encode real patterns into density operators and we have investigated how different encodings yield different classification performances. Throughout this paper we focus on an encoding that is defined by means of the inverse of the standard stereographic projection [21].

Let $X=\left(x^{1},\ldots ,x^{d}\right)\in R^{d}$ be a *d*-dimensional vector. We map the vector $X\in R^{d}$ into a vector $X^{\prime }\in R^{d+1}$ as follows: $X^{\prime }=\alpha \;\left(2x^{1},\ldots ,2x^{d},\sum_{i=1}^{d}{\left(x^{i}\right)}^{2}-1\right)$ where *α* is a normalization factor given by $\alpha =\frac{1}{\sum_{i=1}^{d}{\left(x^{i}\right)}^{2}+1}.$

Now we define the encoded density pattern as: *ρ_X_* = (*X*′)^†^ · (*X*′) Hence, this encoding maps real *d*-dimensional vectors *X* into a (*d* + 1)-dimensional pure state *ρ*_*X*_. This is the encoding that will be used in the experiment which is detailed in Section 4.

### 2.2 Classification

As stated, the main purpose of this work is to provide an original and convenient quantum-inspired classifier. By *quantum-inspired* we mean a classification process that is implemented by a classical computer on macroscopical objects, but which employs and exploits of quantum theory. Some variants of a quantum counterpart of the commonly-used standard *Nearest Mean Classifier* (NMC)—named *Quantum Nearest Mean Classifier* (QNMC)—have recently been analysed. Specifically, the QNMC has been applied to some real and some artificial datasets, obtaining a better performance (in terms of the accuracy of the process and other relevant statistical quantities) compared to the standard NMC. Interestingly, an experiment carried out on a real biomedical dataset provided surprising and highly promising results [19].

As indicated above, the classification process is clearly empirical and it is often hard (or impossible) to determine the superiority of one model over another. In the following sections, we introduce another quantum-inspired classifier and we compare its performances with respect to the QNCM and to some standard classical classifier.

### 2.3 Decoding

The encoding defined above is based on invertible functions that univocally map real vectors onto density operators. After the classification process, rather than applying inverse encoding to each density operator in order to retrieve the original real vector, it would seem more expedient to employ the corresponding label that has been attributed by the classification process. However, having assigned labels for each density pattern of the test set, the decoding turns out to be a non-essential step.

## 3 The Helstrom distance-inspired classifier

In this section we describe a quantum-style classification process for binary classification based on the Helstrom measurement (see [22, 23]), which was initially introduced by Helstrom in a seminal work that addressed the following question: “Suppose to deal with an unknown quantum state drawn from an ensemble of possible pure states where each state is labeled with respect to the class they came from. How well can we predict the class of this unknown quantum state?” [24]. This problem is generally known as the *quantum state discrimination problem* or *quantum classification problem* [7] when referred to machine learning. The answer clearly depends on a number of factors—e.g. crucially, on the amount of available information and on the way this quantity of information might be improved. Unlike in the classical case, in quantum information multiple copies of a quantum state provide more information about it than that is encoded in a single copy thereof. We shall exploit this property in order to improve the classification process inspired by Helstrom’s construct.

Let *ρ*_1_, …, *ρ*_*k*_ be density operators and let associate to each *ρ*_*i*_ operator an *a priori* probability *p*_*i*_. The distribution of the a priori probabilities is determined empirically and in a contextual manner. Let us define a state as a pair {*ρ*, *p*}, where *ρ* is a density operator and *p* is its respective *a priori* probability and let us define a set of states as a set of pairs {(*ρ*_1_, *p*_1_), …, (*ρ*_*k*_, *p*_*k*_)} such that $\sum_{i=1}^{k}p_{i}=1$.

Suppose to have a set consisting of two states {(*ρ*_1_, *p*_1_), (*ρ*_2_, *p*_2_)} such that *p*_1_ + *p*_2_ = 1. It is possible to introduce the operator Λ as:

$$\begin{array}{c} \Lambda =p_{1}\rho_{1}-p_{2}\rho_{2}. \end{array}$$ (1)

It is not difficult to realize that Λ (called *Helstrom observable*) is an Hermitian operator (with trace equal to 0 if $p_{1}=p_{2}=\frac{1}{2}$).

Let $\lambda^{+}=\left\{\lambda_{1}^{+},...,\lambda_{m}^{+}\right\}$ be the set of all the eigenvectors of Λ associated with their respective non negative eigenvalues of Λ and let $\lambda^{-}=\left\{\lambda_{1}^{-},...,\lambda_{l}^{-}\right\}$ (with *m* + *l* ≤ *n*) be the set of all the eigenvectors of Λ associated with their respective negative eigenvalues. Let $P^{+}=\sum_{\lambda_{i}\in \lambda^{+}}P_{\lambda_{i}}$ and $P^{-}=\sum_{\lambda_{i}\in \lambda^{-}}P_{\lambda_{i}},$ where $P_{\lambda_{i}}$ is the projector associated with the eigenvector λ_*i*_ that belongs to λ^+^ or to λ^−^. Notice that, since $P^{+}+P^{-}=I$—where I is the identity matrix—then the set {*P*^+^, *P*^−^} is a von Neumann measurement with respect to the Helstrom observable [25]. The definitions of the projectors *P*^+^ and *P*^−^ enable us to present the following result: suppose now we randomly pick a density operator *ρ* within a multiset *S* where each element of *S* can be only *ρ*_1_ or *ρ*_2_ [23, 26]. The probability that one can correctly discriminate whether *ρ* is *ρ*_1_ or *ρ*_2_ has an upper bound given by:

$$\begin{array}{c} P_{guess}^{\left(\rho_{1},\rho_{2}\right)}=p_{1}Tr\left(P^{+}\rho_{1}\right)+p_{2}Tr\left(P^{-}\rho_{2}\right) \end{array}$$ (2)

where the *a priori* probability can be interpreted as the respective frequencies of *ρ*_1_ and *ρ*_2_ over *S*, i.e., the number of occurrences of *ρ*_1_ and, respectively, *ρ*_2_ over the cardinality of *S*.

The quantity $P_{guess}^{\left(\rho_{1},\rho_{2}\right)}$ is generally called the *Helstrom bound* of the error in the discrimination between the two density operators *ρ*_1_ and *ρ*_2_ and it can be seen as a measurement of distinguishability between *ρ*_1_ and *ρ*_2_ [7, 27]. Intuition seems to tell us that *p*_1_*Tr*(*P*^+^*ρ*_1_) represents the conditional probability that, if we pick *ρ* = *ρ*_1_, then *ρ* is correctly identified as *ρ*_1_; the same applies for *p*_2_*Tr*(*P*^−^*ρ*_2_). Clearly, if there is an equal probability that, randomly picking a density operator *ρ*, this *ρ* is *ρ*_1_ or *ρ*_2_, then $p_{1}=p_{2}=\frac{1}{2}$. Given the fact that the accuracy of a classification process is an empirical value (it can change, in principle, for any different run of the process) it is not easy to find a formal correlation between accuracy and $P_{guess}^{\left(\rho_{1},\rho_{2}\right)}$. However, let us consider a binary classification process based on the distinguishability between two centroids *ρ*_1_ and *ρ*_2_ of the two different classes. In this case it is robustly reasonable to assume that a high value of $P_{guess}^{\left(\rho_{1},\rho_{2}\right)}$ (i.e., a high probability of distinguishing between the two centroids) should be related to a “good” performance of the classifier. Further, in order to optimize the value of $P_{guess}^{\left(\rho_{1},\rho_{2}\right)}$, it will be useful to recall the fact that providing additional copies of quantum states makes it possible to obtain a lowered error in the discrimination probability [28].

To obtain a significantly lower error in the state discrimination, let us now describe the new quantum-style classification procedure, inspired by the Helstrom model referred to above. Hereafter, we will consider binary classification (i.e., with two classes only) and we will assume that the labels *l*_*m*_ ∈ {+, −}, for all *m* ∈ {1, …, *M*}.

Given a training dataset *S*_*tr*_ = {(*X*_1_, *l*_1_), …, (*X*_*M*_, *l*_*M*_)} we can define the *positive class* $S_{tr}^{+}$ and the *negative class* $S_{tr}^{-}$ of *S*_*tr*_ as follows:

$$\begin{array}{c} S_{tr}^{+}=\left\{\left(X_{i},l_{i}\right)\in S_{tr}:l_{i}=+\right\}\;\;\text{and}\;\;S_{tr}^{-}=\left\{\left(X_{i},l_{i}\right)\in S_{tr}:l_{i}=-\right\}. \end{array}$$ (3)

$S_{tr}^{+}$ ($S_{tr}^{-}$, respectively) is the set of all patterns of the training dataset belonging to the class labeled by + (−, respectively).

By *M*^+^ (*M*^−^, respectively) we will denote the cardinality of $S_{tr}^{+}$ (of $S_{tr}^{-}$, respectively). Clearly, *M*^+^ + *M*^−^ = *M*. Following standard procedure, in order to introduce a quantum version of the classification process, we first need to encode any real vector *X*_*i*_ in terms of a density operator $\rho_{X_{i}}$. After using one encoding from real vectors to density operators (by means of the stereographic encoding outlined in Section 2.1 or the informative encoding introduced in [17]), it is possible to establish the definitions for the quantum centroids in the positive and the negative classes.

Let $QS_{tr}=\{\left\{\rho_{X_{1}},l_{1}\right\},\ldots ,\left\{\rho_{X_{M}},l_{M}\right\}\}$ be a quantum training dataset of density patterns. The *Quantum Centroids* for the positive and the negative class are given, respectively, by:

$$\begin{array}{c} \rho^{+}=\frac{1}{M^{+}}\sum_{i\in \{m:l_{m}=+\}}\rho_{X_{i}}\;\text{and}\;\rho^{-}=\frac{1}{M^{-}}\sum_{i\in \{m:l_{m}=-\}}\rho_{X_{i}}. \end{array}$$ (4)

Notice that the quantum centroids are generally mixed states and clearly do not correspond to the density patterns obtained by encoding the centroids of the original dataset. Interestingly, the expressions of the quantum centroids do not remain invariant if the coordinates of the dataset are rescaled. If we then rescale all the features of a given dataset by a real parameter *t*, the new centroid *C*_*t*_ of the rescaled dataset is obtained by rescaling the coordinates of the original centroid *C*. However, it seems quite clear that in general, the encoding process does not preserve the centroid, a fact that significantly affects the classification process. In [21] we have shown that the failure of invariance under rescaling plays the role of an asset: indeed, the rescaling factor *t* can be used as a free parameter to optimize the accuracy of the classification process.

Given a training dataset, it is possible to define the *quantum Helstrom observable* associated to it as:

$$\begin{array}{c} \Lambda_{Q}=\frac{M^{+}}{M}\rho^{+}-\frac{M^{-}}{M}\rho^{-}. \end{array}$$ (5)

This expression is analogous to Eq 1, where the *a priori* probability is obtained as the frequencies of the elements belonging to the positive and negative classes, respectively, over the entire training dataset.

Let $\lambda_{Q}^{+}=\left\{\lambda_{Q1}^{+},...,\lambda_{Qm}^{+}\right\}$ be the set of all eigenvectors of Λ_*Q*_ associated with the non negative eigenvalues and let $\lambda_{Q}^{-}=\left\{\lambda_{Q1}^{-},...,\lambda_{Ql}^{-}\right\}$ (with *m* + *l* ≤ *n*) be the set of all eigenvectors of Λ_*Q*_ associated with the negative eigenvalues. Let $P_{Q}^{+}=\sum_{\lambda_{Qi}\in \lambda_{Q}^{+}}P_{\lambda_{Qi}}$ and $P_{Q}^{-}=\sum_{\lambda_{Qi}\in \lambda_{Q}^{-}}P_{\lambda_{Qi}},$ where $P_{\lambda_{Qi}}$ is the projector associated with the eigenvector λ_*Qi*_ that belongs to $\lambda_{Q}^{+}$ or to $\lambda_{Q}^{-}$, respectively. The *positivity* and *negativity* of the eigenvalues of the Helstrom observables should not be confused with the *positivity* and *negativity* of the classes. More precisely, in $P_{Q}^{+}$ and $P_{Q}^{-}$, the signs + and − are referred to the sign of the eigenvalues of the Helstrom observable; on the other hand, in the expression of the centroids *ρ*^+^ and *ρ*^−^, the signs + and − are referred to the positive and negative class, respectively.

Given an arbitrary pattern *X* that belongs to the *test dataset*, the classification of *X* as belonging to the positive or to the negative class is dictated by the following classification function.

Let *ρ*_*X*_ be the density pattern associated with the *d*-dimensional real vector *X* (obtained by means of encoding). It is possible to define a *Helstrom Quantum Centroid* classifier (HQC) as follows:

$$\begin{array}{c} HQC\left(\rho_{X}\right)=\{\begin{array}{cc} + & \;\;\text{if}\;\;\;\;\;Tr\left(\rho_{X}P_{Q}^{+}\right)\geq Tr\left(\rho_{X}P_{Q}^{-}\right);\\ - & \;\;\text{otherwise.} \end{array} \end{array}$$ (6)

Finally, in this case and referring to Eq 2 the Helstrom bound will be reasonably defined as:

$$\begin{array}{c} P_{guess}^{\left(\rho^{+},\rho^{-}\right)}=\frac{M^{+}}{M}Tr\left(\rho^{+}P_{Q}^{+}\right)+\frac{M^{-}}{M}Tr\left(\rho^{-}\right)P_{Q}^{-}. \end{array}$$ (7)

### 3.1 The HQC with copies

We shall now describe a variant of HQC that essentially consists in making *n* copies of each density pattern of the dataset. This procedure is consistent due to the fact that, unlike in the classical case, in quantum information the state *ρ*⊗ … ⊗*ρ* (the *n*-fold tensor product of *ρ*) is generally more informative than the single state *ρ*.

Accordingly, we proceed as follows both for the training and for the test datasets.

Given a training dataset *S*_*tr*_ and its respective quantum training dataset $QS_{tr}=\{\left\{\rho_{X_{1}},l_{1}\right\},\ldots ,\left\{\rho_{X_{M}},l_{M}\right\}\}$ let us define the *n*-quantum training dataset (⊗^*n*^)*QS*_*tr*_ as follows:

$$\begin{array}{c} \left({⊗}^{n}\right)QS_{tr}=\{\left\{{⊗}^{n}\rho_{X_{1}},l_{1}\right\},\ldots ,\left\{{⊗}^{n}\rho_{X_{M}},l_{M}\right\}\}, \end{array}$$ (8)

where by ${⊗}^{n}\rho_{X_{i}}$ we denote the *n*-fold tensor product of $\rho_{X_{i}}$.

Thus, according to Eqs 1 and 4 we can define the *n*-quantum centroids (⊗^*n*^)*ρ*^+^ and (⊗^*n*^)*ρ*^−^ as well as the *n*-quantum Helstrom observable (⊗^*n*^)Λ_*Q*_ with its associated von Neumann measurement $\left\{\left({⊗}^{n}\right)P_{Q}^{+},\left({⊗}^{n}\right)P_{Q}^{-}\right\}$. Let us remark that in general (⊗^*n*^)*ρ*^+^ ≠ ⊗^*n*^ *ρ*^+^, i.e., the centroid of the density patterns of (⊗^*n*^)*QS*_*tr*_ labeled by + is not the *n*-fold tensor product of the centroid of the density patterns of *QS*_*tr*_ labeled by +. Similarly holds for *ρ*^−^, Λ_*Q*_, $P_{Q}^{+}$ and $P_{Q}^{-}$.

We can define the *n*-Helstrom Quantum Centroid classifier ((⊗^*n*^)*HQC*) in a similar way to Eq 6.

Let *ρ*_*X*_ be the density pattern associated with the *d*-dimensional real vector *X*. Then,

$$\begin{array}{c} \left({⊗}^{n}\right)HQC\left(\rho_{X}\right)=\{\begin{array}{cc} + & \;\;\text{if}\;\;\;\;\;Tr\left({⊗}^{n}\rho_{X}\left(\left({⊗}^{n}\right)P_{Q}^{+}\right)\right)\geq Tr({⊗}^{n}\rho_{X}\left(\left({⊗}^{n}\right)P_{Q}^{-}\right);\\ - & \;\;\text{otherwise.} \end{array} \end{array}$$ (9)

and the *n-Helstrom bound* can be generalized as follows:

$$\begin{array}{c} P_{guess}^{\left(\left({⊗}^{n}\right)\rho^{+},\left({⊗}^{n}\right)\rho^{-}\right)}=\frac{M^{+}}{M}Tr\left(\left({⊗}^{n}\right)\rho^{+}\left({⊗}^{n}\right)P_{Q}^{+}\right)+\frac{M^{-}}{M}Tr\left(\left({⊗}^{n}\right)\rho^{-}\left({⊗}^{n}\right)P_{Q}^{-}\right). \end{array}$$ (10)

It can be proved (see Appendix A) that, in the simple special case where all the density patterns (belonging to both training and test datasets) are diagonal, with dimension two and conditions are such that *M*^+^ = *M*^−^, then the value of the *n*-Helstrom bound decreases by making a copy for each density pattern. A systematic and complete theoretical analysis is yet to be undertaken and shall be left to a future study.

In the next section we show some results based on the application of this classifier to different two-classes datasets. The outcomes of our experiments show that on average the (⊗^*n*^)HQC classifier outperforms the large number of commonly used classifiers.

## 4 The experiment

In this section we show some significant improvement to the performances of HQC and (⊗^*n*^)HQC with respect to a large set of classifiers that generally perform well for different kinds of datasets. Given the nature of HQC, we restrict the experimental setup to binary datasets, i.e., datasets with only two classes.

Depending on the particular distribution of the dataset, it is possible that a pattern belonging to a given class is incorrectly classified. For an arbitrary pattern (*X*_*i*_, λ_*i*_), four cases are possible:

- *X*_*i*_ is a *true positive* (TP) object: the pattern (*X*_*i*_, λ_*i*_) belongs to the positive class $S_{\text{tr}}^{+}$, and it is correctly classified, i.e., *Cl*(*X*_*i*_) = +
- *X*_*i*_ is a *true negative* (TN) object: the pattern (*X*_*i*_, λ_*i*_) belongs to the negative class $S_{\text{tr}}^{-}$, and it is correctly classified, i.e., *Cl*(*X*_*i*_) = −
- *X*_*i*_ is a *false positive* (FP) object: the pattern (*X*_*i*_, λ_*i*_) belongs to negative class $S_{\text{tr}}^{-}$, but it is incorrectly classified, i.e., *Cl*(*X*_*i*_) = +
- *X*_*i*_ is a *false negative* (FN) object: the pattern(*X*_*i*_, λ_*i*_) belongs to positive class $S_{\text{tr}}^{+}$, but it is incorrectly classified, i.e., *Cl*(*X*_*i*_) = −

Then, by applying the classifier to the test set, it is possible to assess its performance by considering the following significant statistical quantities:

- *Accuracy*: $Ac=\frac{\#TP+\#TN}{\#TP+\#TN+\#FP+\#FN}$
- *Sensitivity*: $Se=\frac{\#TP}{\#TP+\#FN}$
- *Specificity*: $Sp=\frac{\#TN}{\#TN+\#FP}$
- *Balanced Accuracy*: $Ba=\frac{1}{2}\left(\frac{\#TP}{\#TP+\#FN}+\frac{\#TN}{\#FP+\#TN}\right)$
- *Precision*: $Pr=\frac{\#TP}{\#TP+\#FP}$
- *F*–*measure*: $F-m=\frac{2Pr\cdot Se}{Pr+Se}$
- *Cohen’s* *k* *parameter*: $k=\frac{Pr(a)-Pr(e)}{1-Pr(e)}$ where $Pr\left(a\right)=\frac{\#TP+\#TN}{M_{+}^{\prime }+M_{-}^{\prime }}$ and $Pr\left(e\right)=\frac{\left(\#TP+\#FP)(\#TP+\#FN)+(\#FP+\#TN)(\#TN+\#FN\right)}{{\left(M_{+}^{\prime }+M_{-}^{\prime }\right)}^{2}}$.
  Notice that −1 ≤ *k* ≤ 1; intuitively, the case *k* = 1 corresponds to a perfect classification (error *E* = 0); on the other hand, the case *k* = −1 results in an entirely wrong classification (error *E* = 1).

In the following section we apply HQC to several datasets and we compare its performances with other commonly used classifiers, showing the marked average superiority of HCQ compared to all the other classifiers.

### 4.1 Experimental setup and methodology

In order to assess the performances of HQC and (⊗^*n*^)HQC, we first apply these classifiers to fourteen different datasets and we evaluate all the statistical quantities described above. Then, having applied other commonly used (and generally well-performing) classifiers to the same datasets, we then compare the resulting outcomes.

The datasets we are dealing with are extracted from the PMLB repository (Penn Machine Learning Benchmark) [29]. This repository includes datasets that typically take one of three forms. The first is accessible, well-studied *real-world data*, taken from different real-world problem domains of interest. The second is *simulated data*, or data that have been artificially generated, often to ‘look’ like real-world data, but with known, underlying patterns. The third form is *toy data*, which we define here as data that is also artificially generated with a known embedded pattern but without an emphasis on representing real-world data. We consider all of these kinds of datasets. More precisely, we have run on these 14 datasets: *Banana*, *Prnn synth*, *Analcatdata aids*, *Haberman*, *Moon*, *Lupus*, *Gaussian*, *Titanic*, *Analcatdata boxing1*, *Analcatdata asbestos*, *Appendicitis*, *Analcatdata boxing2*, *Hill Valley (with noise)*, *Hill Valley (without noise)*.

We have applied HQC on these datasets by making copies of each element of a given dataset as described in 3.1. Next, using *HelstromQuantumCentroid#* we indicate the application of HQC with # number of copies. In particular, HQC has been run for # ∈ {1, …, 4}. The other classifiers that we applied to the datasets listed above, are: *BernoullyNB*, *Logistic Regression*, *Gaussian NB*, *K-Neighbors Classifier*, *Random Forest Classifier*, *Ada Boost Classifier*, *Nearest Centroid*, *Linear Discriminant Analysis*, *Extra Trees Classifier*, *Gradient Boosting Classifier*, and *QNMC*.

**Fig 1:**
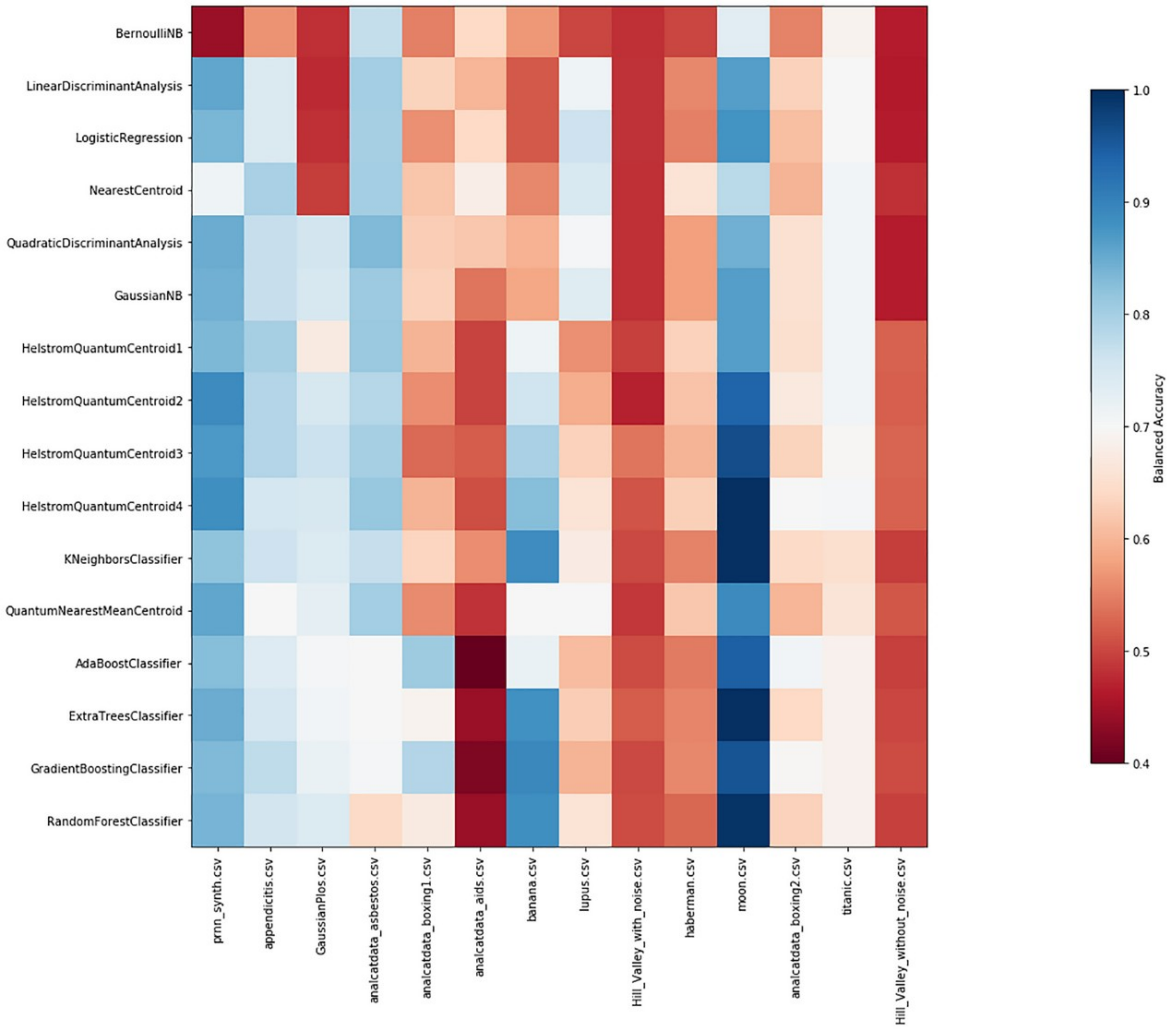
Biclustering of the 16 classifiers and 14 datasets according to Balanced Accuracy.

**Fig 2:**
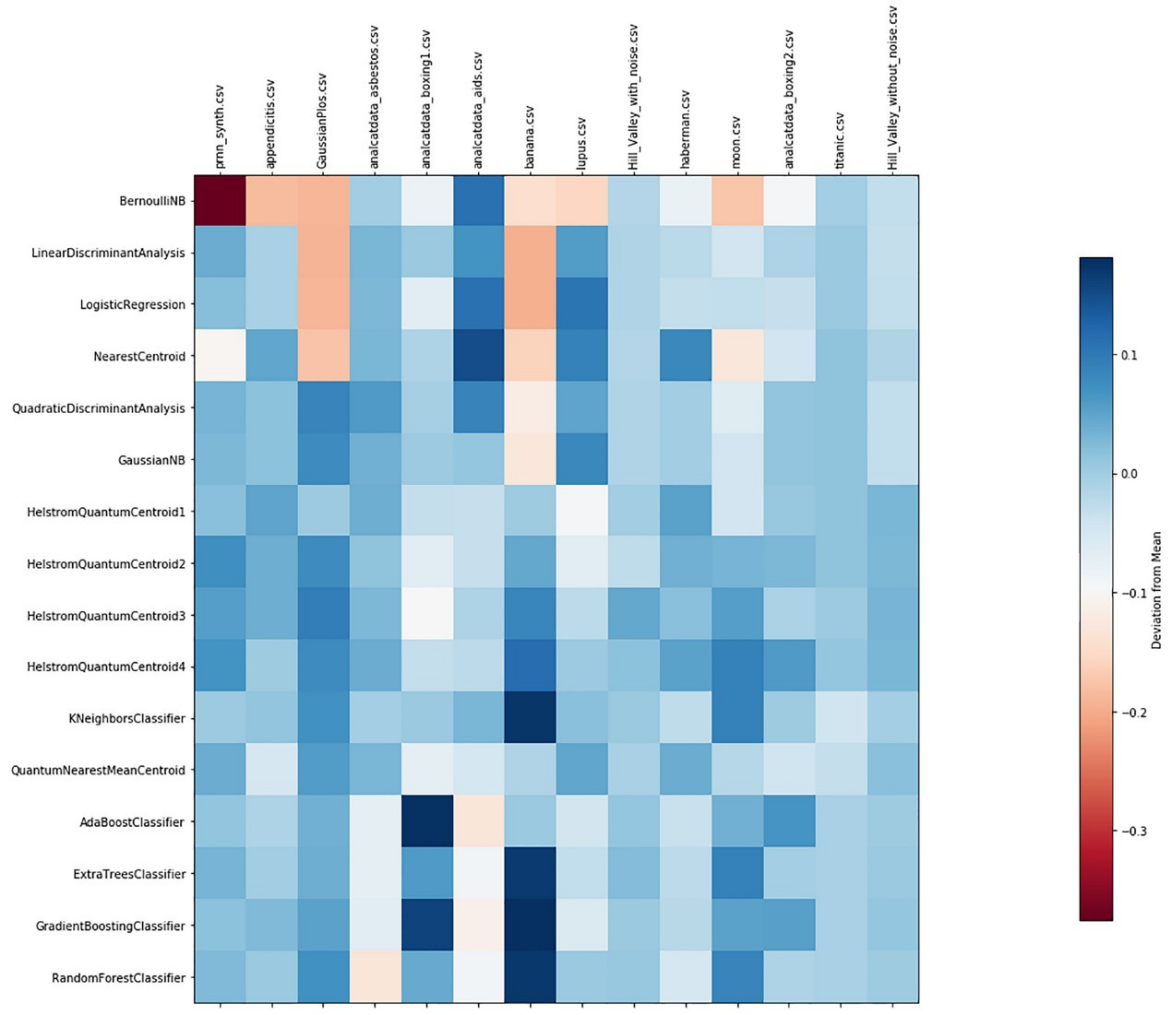
Deviation from the mean Balanced Accuracy across all 16 classifiers.

**Fig 3:**
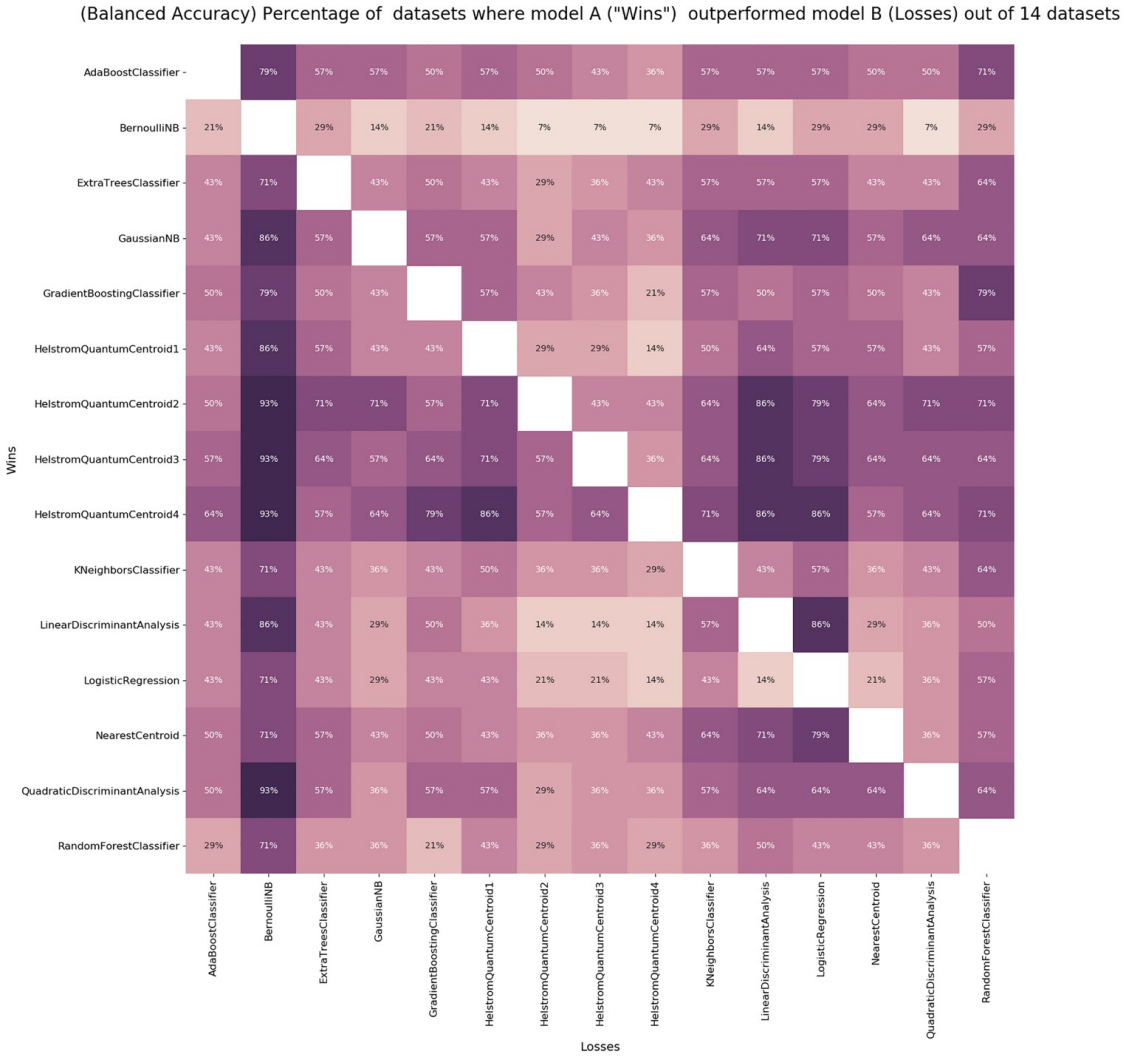
When a classifier A outperforms a classifier B according to the Balanced Accuracy.

The experimental procedure was repeated identically for each dataset and was implemented by a standard version of Python. The experiment essentially involves following the four steps described here:

1. Tuning: we run the HQC algorithm by considering the full dataset as a training set. However, before each encoding, we rescale the dataset factor *t*, as discussed in Section 4. We range the rescaling factor *t* along consecutive subintervals of length 0.5 within the interval (0.1, 10). For each dataset *D*_*i*_, we obtain the *optimal* value of *t*_*i*_, i.e., the value of *t*_*i*_ that produces the best Balanced Accuracy of the HQC classifier (with respect to the given interval). We rescale each dataset *D*_*i*_ by *t*_*i*_ and we encode the dataset using the encoding described in Section 2.1. Note that the action of the rescaling factor *t* does not affect the performances of all the other (classical) standard classifiers.
2. Cross validation: for each dataset we randomly pick 80% of the dataset as the training set and we effect the classification on the rest of the dataset. Once the same procedure has been repeated 10 times, we present all statistical values introduced at the beginning of Section 5.
3. Local Comparison: we repeat the same steps described above for all the 14 datasets and for all the classifiers. In particular, we apply HQC with 1,2,3 and 4 copies. In so doing, we obtain a comparison table for each dataset. See Tables A—O in S1 File.
4. Global Comparison: by extracting data related to the Balanced Accuracy from Tables A-O in S1 File, we can provide a comparison between and among all the classifiers. In particular, in Fig 1, we resume the value of the mean Balanced Accuracy of each classifier and for any dataset. In Fig 2, we indicate the respective standard deviations. In Fig 3, we depict a two-by-two comparison between all the classifiers. In particular, for each classifier in the left-hand column we count the success rate achieved by comparing the Balance Accuracy obtained by applying it on each dataset with the Balance accuracy obtained by applying the other classifiers named in the line below the picture. Finally, in Table O in S1 File we summarize the Average Success Rate of each of the classifiers over all datasets.

### 4.2 Discussion

The crucial result is shown in Fig 3 and Table O in S1 File: over all the 14 datasets HQC is, on average, the classifier that consistently exhibits the best performance. In particular, HQC with 4 copies has an Average Success Rate equal to 72.8%, while the first standard classifier below HQC is the Gaussian NB (Gaussian Naive Bayes) whose Average Success Rate is -only- equal to 58%. Further, by looking at Fig 1, it is possible to see that HQC with 4 copies is one of the best-performing classifiers (in terms of Balanced Accuracy) for almost all the datasets (except for the *analcatdata asbestos* and *lupus* datasets). We can also see that, in general, an increase in the number of copies produces an improvement in all the statistical quantities: Tables A-O in S1 File demonstrate the high efficiency of the HQC classifier not only with respect to the Balanced Accuracy but, proportionally, also with respect to all the other statistical quantities. Also, the values of these quantities—on average—improve more and more as the number of the copies increases.

## 5 Concluding remarks and further developments

In this paper we have introduced an innovative technique—inspired by the formalism of quantum theory—to design a new kind of supervised classifier (HQC) and we have provided a full comparison of the performances of this new classifier with respect to many others (linear and non linear) frequently used classifiers. The HQC proves to be superior, on average, compared to all the statistical parameters we considered. As a result, we believe that the potential of the quantum formalism as an application for classification processes in the classical context is extremely promising.

However, further research of both a theoretical and applied nature, is needed. From a theoretical viewpoint, special attention will be devoted to investigating the encoding step in order to find, for any dataset (or for specific classes of datasets) the *most suitable* encoding procedure, i.e., the encoding that provides the best performance of the HQC classifier. Another theoretical investigation should be devoted to generalizing HQC in such a way as to also use the classifier for *n*-ary classification (not only binary classification). Finally, as we have seen, an increase in the number of copies of the HQC classifier, frequently benefits the classification process. However, this increase provides a non-negligible computation cost that prevents us from running the algorithm with more than four copies in cases of large datasets or in cases of many-features vectors. Hence, this technical problem also deserves further attention.

On the other hand, with regard to practical implementation, previous works discussed above [19] have pointed to possible applications of these kinds of approaches in the biomedical field. Anyway, these non-standard applications are, though, still at an initial stage. An in-depth interdisciplinary investigation will be carried on in this direction: these early achievements might well be extended to different contexts of application, including pattern recognition, fingerprint recognition and, in short, all the contexts where the classification procedure plays a crucial role.

## Appendix A

Let us consider two classes of the same cardinality *C*_1_ = {*ρ*_*i*_}_*i*=1,⋯,*n*_ and *C*_2_ = {*σ*_*i*_}_*i*=1,⋯,*n*_ of 2 × 2- diagonal density matrices, where *diag*[*ρ*_*i*_] = [1 − *r*_*i*_, *r*_*i*_] and *diag*[*σ*_*i*_] = [1 − *s*_*i*_, *s*_*i*_] and where the non trivial cases 0 < *r*_*i*_, *s*_*i*_ < 1 are considered. Let us indicate by *ρ* and *σ* the centroids of the classes *C*_1_ and *C*_2_, respectively, and let us suppose that *ρ* ≠ *σ*. By referring to Eq 10, we show that $P_{guess}^{\left(\left({⊗}^{2}\right)\rho ,\left({⊗}^{2}\right)\sigma \right)}\geq P_{guess}^{\left(\rho ,\sigma \right)}$.

By Eq 4, we have that $diag\left[\rho \right]=\frac{1}{n}\left[n-\sum_{i=1}^{n}r_{i},\sum_{i=1}^{n}r_{i}\right]$ and $diag\left[\sigma \right]=\frac{1}{n}\left[n-\sum_{i=1}^{n}s_{i},\sum_{i=1}^{n}s_{i}\right]$. By Eq 5 we obtain: *diag*[Λ_*Q*_] = [*α*, −*α*], where $\alpha =\frac{\sum_{i=1}^{n}\left(s_{i}-r_{i}\right)}{2n}$. Let us notice that *α* can not be zero because *ρ* ≠ *σ*. The values {*α*, −*α*} are the eigenvalues of Λ_*Q*_ and the respective eigenvectors are: {(1, 0)^†^, (0, 1)^†^}. Without loss of generality, let us suppose that *α* > 0. In this case, $P_{Q}^{+}={\left(1,0\right)}^{†}\left(1,0\right)$ and analogously $P_{Q}^{-}={\left(0,1\right)}^{†}\left(0,1\right)$; hence, by a straightforward calculation we obtain that $P_{guess}^{\left(\rho ,\sigma \right)}=\frac{1}{2}Tr\left(P_{Q}^{+}\rho \right)+\frac{1}{2}Tr\left(P_{Q}^{-}\sigma \right)=\frac{1}{2}+\alpha$. Now we proceed in order to evaluate the quantity $P_{guess}^{\left(\left({⊗}^{2}\right)\rho ,\left({⊗}^{2}\right)\sigma \right)}$.

Let us consider the two classes $C_{1}^{\left(2\right)}={\left\{\rho_{i}⊗\rho_{i}\right\}}_{i=1,...,n}$ and $C_{2}^{\left(2\right)}={\left\{\sigma_{i}⊗\sigma_{i}\right\}}_{i=1,...,n}$. Let (⊗^2^)*ρ* and (⊗^2^)*σ* be the centroids of $C_{1}^{\left(2\right)}$ and $C_{2}^{\left(2\right)}$, respectively. We have that $diag\left[\left({⊗}^{2}\right)\rho \right]=\frac{1}{n}\left[\sum_{i=1}^{n}{\left(1-r_{i}\right)}^{2},\sum_{i=1}^{n}r_{i}\left(1-r_{i}\right),\sum_{i=1}^{n}r_{i}\left(1-r_{i}\right),\sum_{i=1}^{n}r_{i}^{2}\right]$. Analogously for *diag*[(⊗^2^)*σ*]. By defining $\left({⊗}^{2}\right)\Lambda_{Q}=\frac{1}{2}\left(\left({⊗}^{2}\right)\rho -\left({⊗}^{2}\right)\sigma \right)$, it is easy to see that the eigenvalues of (⊗^2^)Λ_*Q*_ are {2*α* − *β*, *β* − *α*, *β* − *α*, −*β*} (where *α* has been previously defined and $\beta =\frac{\sum_{i=1}^{n}\left(s_{i}^{2}-r_{i}^{2}\right)}{2n}$) and the respective eigenvectors are {(1, 0, 0, 0)^†^, (0, 1, 0, 0)^†^, (0, 0, 1, 0)^†^, (0, 0, 0, 1)^†^}. For the sake of simplicity, we also call the eigenvalues of (⊗^2^)Λ_*Q*_ as {*μ*_1_, *μ*_2_, *μ*_3_, *μ*_4_}. Now, reminding that we have assumed *α* > 0, from the assumption that 0 < *r*_*i*_, *s*_*i*_ < 1, it trivially follows that 0 < *β* < 2*α*. Hence, only the following cases are possible:

1. 0 < *β* < *α* ⇒ *μ*_1_ > 0, *μ*_2_ = *μ*_3_ < 0, *μ*_4_ < 0;
2. *α* < *β* < 2*α* ⇒ *μ*_1_ > 0, *μ*_2_ = *μ*_3_> 0, *μ*_4_ < 0;
3. *β* = *α* ⇒ * μ*_1_ > 0, *μ*_2_ = *μ*_3_ = 0, *μ*_4_ < 0.

Case 1) In this case is $\left({⊗}^{2}\right)P_{Q}^{+}=(\begin{array}{cccc} 1 & 0 & 0 & 0\\ 0 & 0 & 0 & 0\\ 0 & 0 & 0 & 0\\ 0 & 0 & 0 & 0 \end{array})$ and $\left({⊗}^{2}\right)P_{Q}^{-}=(\begin{array}{cccc} 0 & 0 & 0 & 0\\ 0 & 1 & 0 & 0\\ 0 & 0 & 1 & 0\\ 0 & 0 & 0 & 1 \end{array})$.

Hence, $P_{guess}^{\left(\left({⊗}^{2}\right)\rho ,\left({⊗}^{2}\right)\sigma \right)}=\frac{1}{2n}\sum_{i=1}^{n}\left(1+r_{i}^{2}-2r_{i}+2s_{i}-2s_{i}^{2}+s_{i}^{2}\right)=\frac{1}{2}+2\alpha -\beta$ and $P_{guess}^{\left(\left({⊗}^{2}\right)\rho ,\left({⊗}^{2}\right)\sigma \right)}-P_{guess}^{\left(\rho ,\sigma \right)}=\alpha -\beta$ that is always positive, because *α* > 0 and 0 < *β* < *α*.

Case 2) In this case is $\left({⊗}^{2}\right)P_{Q}^{+}=(\begin{array}{cccc} 1 & 0 & 0 & 0\\ 0 & 1 & 0 & 0\\ 0 & 0 & 1 & 0\\ 0 & 0 & 0 & 0 \end{array})$ and $\left({⊗}^{2}\right)P_{Q}^{-}=(\begin{array}{cccc} 0 & 0 & 0 & 0\\ 0 & 0 & 0 & 0\\ 0 & 0 & 0 & 0\\ 0 & 0 & 0 & 1 \end{array})$.

Hence, $P_{guess}^{\left(\left({⊗}^{2}\right)\rho ,\left({⊗}^{2}\right)\sigma \right)}=\frac{1}{2}+\beta$ and $P_{guess}^{\left(\left({⊗}^{2}\right)\rho ,\left({⊗}^{2}\right)\sigma \right)}-P_{guess}^{\left(\rho ,\sigma \right)}=\beta -\alpha$ that is always positive because *α* < *β* < 2*α*.

Finally, Case 3) follows the same scheme of Case 2) and it allows us to obtain that $P_{guess}^{\left(\left({⊗}^{2}\right)\rho ,\left({⊗}^{2}\right)\sigma \right)}=P_{guess}^{\left(\rho ,\sigma \right)}$.

## Supporting information

S1 File Table A. Banana dataset (with #*n* number of copies). Table B. Prnn-synth dataset (with #*n* number of copies). Table C. Analcatdata aids dataset (with #*n* number of copies). Table D. Haberman dataset (with #*n* number of copies). Table E. Moon dataset (with #*n* number of copies). Table F. Lupus dataset (with #*n* number of copies). Table G. GaussianPlos dataset (with #*n* number of copies). Table H. Titanic dataset (with #*n* number of copies). Table I. Analcatdata-boxing1 dataset (with #*n* number of copies). Table J. Analcatdata-asbestos dataset (with #*n* number of copies). Table K. Appendicitis dataset (with #*n* number of copies). Table L. Analcatdata-boxing2 dataset (with #*n* number of copies). Table M. Hill-Valley-with-noise dataset (with #*n* number of copies). Table N. Hill-Valley-without-noise dataset (with #*n* number of copies). Table O. Average Success Rate.
(PDF)
