# Supplementary material for: A new quantum approach to binary classification
Source: PLoS One. 2019 May 9;14(5):e0216224. doi: 10.1371/journal.pone.0216224 (PMC6508868; doi:10.1371/journal.pone.0216224)
Supplement: S1 File — Table A. Banana dataset (with #n number of copies). Table B. Prnn-synth dataset (with #n number of copies). Table C. Analcatdata aids dataset (with #n number of copies). Table D. Haberman dataset (with #n number of copies). Table E. Moon dataset (with #n number of copies). Table F. Lupus dataset (with #n number of copies). Table G. GaussianPlos dataset (with #n number of copies). Table H. Titanic dataset (with #n number of copies). Table I. Analcatdata-boxing1 dataset (with #n number of copies). Table J. Analcatdata-asbestos dataset (with #n number of copies). Table K. Appendicitis dataset (with #n number of copies). Table L. Analcatdata-boxing2 dataset (with #n number of copies). Table M. Hill-Valley-with-noise dataset (with #n number of copies). Table N. Hill-Valley-without-noise dataset (with #n number of copies). Table O. Average Success Rate. (PDF) [file pone.0216224.s001.pdf]

Tables

Classifier	Accuracy	Sensitivity	Specificity	Balanced Accuracy	Precision	F-measure	Cohen's k	Helstrom Error
BernoulliNB	0.57 ± 0.02	0.59 ± 0.02	0.55 ± 0.03	0.57 ± 0.02	0.55 ± 0.02	0.52 ± 0.02	0.14 ± 0.04	
LogisticRegression	0.56 ± 0.02	0.13 ± 0.02	0.91 ± 0.04	0.52 ± 0.02	0.2 ± 0.02	0.55 ± 0.13	0.04 ± 0.05	
GaussianNB	0.61 ± 0.01	0.31 ± 0.03	0.86 ± 0.02	0.59 ± 0.01	0.42 ± 0.03	0.64 ± 0.03	0.18 ± 0.03	
KNeighborsClassifier	0.89 ± 0.01	0.86 ± 0.02	0.91 ± 0.02	0.88 ± 0.01	0.87 ± 0.02	0.88 ± 0.02	0.77 ± 0.03	
RandomForestClassifier	0.88 ± 0.01	0.88 ± 0.02	0.89 ± 0.02	0.88 ± 0.01	0.87 ± 0.01	0.86 ± 0.02	0.76 ± 0.02	
AdaBoostClassifier	0.72 ± 0.01	0.64 ± 0.03	0.79 ± 0.03	0.72 ± 0.01	0.67 ± 0.02	0.72 ± 0.02	0.44 ± 0.03	
NearestCentroid	0.55 ± 0.02	0.58 ± 0.03	0.53 ± 0.03	0.55 ± 0.02	0.54 ± 0.02	0.5 ± 0.02	0.11 ± 0.04	
LinearDiscriminantAnalysis	0.56 ± 0.02	0.13 ± 0.02	0.91 ± 0.04	0.52 ± 0.02	0.2 ± 0.02	0.55 ± 0.13	0.04 ± 0.05	
QuadraticDiscriminantAnalysis	0.62 ± 0.02	0.35 ± 0.03	0.84 ± 0.03	0.59 ± 0.02	0.45 ± 0.03	0.64 ± 0.05	0.2 ± 0.05	
ExtraTreesClassifier	0.88 ± 0.01	0.87 ± 0.02	0.89 ± 0.01	0.88 ± 0.01	0.87 ± 0.01	0.87 ± 0.01	0.77 ± 0.02	
GradientBoostingClassifier	0.9 ± 0.01	0.85 ± 0.02	0.94 ± 0.01	0.89 ± 0.01	0.88 ± 0.01	0.92 ± 0.01	0.79 ± 0.02	
QuantumNearestMeanCentroid	0.7 ± 0.01	0.7 ± 0.03	0.7 ± 0.02	0.7 ± 0.02	0.65 ± 0.02	0.68 ± 0.02	0.4 ± 0.03	
HelstromQuantumCentroid1	0.7 ± 0.02	0.81 ± 0.03	0.61 ± 0.02	0.71 ± 0.02	0.63 ± 0.02	0.71 ± 0.02	0.42 ± 0.04	0.36 ± 0.0
HelstromQuantumCentroid2	0.78 ± 0.01	0.56 ± 0.03	0.96 ± 0.02	0.76 ± 0.02	0.92 ± 0.03	0.69 ± 0.02	0.54 ± 0.03	0.33 ± 0.0
HelstromQuantumCentroid3	0.8 ± 0.01	0.76 ± 0.02	0.84 ± 0.02	0.8 ± 0.01	0.79 ± 0.02	0.78 ± 0.01	0.6 ± 0.01	0.3 ± 0.0
HelstromQuantumCentroid4	0.83 ± 0.01	0.81 ± 0.02	0.85 ± 0.02	0.83 ± 0.01	0.81 ± 0.03	0.81 ± 0.02	0.66 ± 0.02	0.27 ± 0.0

Table 1. Banana dataset (with $\#n$ number of copies)

Classifier	Accuracy	Sensitivity	Specificity	Balanced Accuracy	Precision	F-measure	Cohen's k	Helstrom Error
BernoulliNB	0.48 ± 0.1	0.57 ± 0.24	0.4 ± 0.23	0.48 ± 0.1	0.51 ± 0.13	0.49 ± 0.11	-0.03 ± 0.2	
LogisticRegression	0.85 ± 0.06	0.9 ± 0.07	0.79 ± 0.14	0.85 ± 0.06	0.86 ± 0.05	0.83 ± 0.1	0.69 ± 0.12	
GaussianNB	0.83 ± 0.07	0.86 ± 0.1	0.81 ± 0.14	0.83 ± 0.07	0.84 ± 0.06	0.83 ± 0.1	0.67 ± 0.13	
KNeighborsClassifier	0.82 ± 0.08	0.81 ± 0.12	0.83 ± 0.12	0.82 ± 0.08	0.82 ± 0.08	0.84 ± 0.11	0.64 ± 0.16	
RandomForestClassifier	0.82 ± 0.04	0.81 ± 0.1	0.83 ± 0.12	0.82 ± 0.04	0.81 ± 0.04	0.84 ± 0.1	0.63 ± 0.09	
AdaBoostClassifier	0.84 ± 0.07	0.88 ± 0.08	0.81 ± 0.13	0.84 ± 0.07	0.85 ± 0.06	0.83 ± 0.1	0.69 ± 0.13	
NearestCentroid	0.71 ± 0.11	0.78 ± 0.1	0.63 ± 0.19	0.71 ± 0.11	0.73 ± 0.09	0.7 ± 0.12	0.42 ± 0.21	
LinearDiscriminantAnalysis	0.84 ± 0.06	0.86 ± 0.1	0.82 ± 0.12	0.84 ± 0.06	0.84 ± 0.06	0.84 ± 0.09	0.68 ± 0.12	
QuadraticDiscriminantAnalysis	0.85 ± 0.06	0.88 ± 0.09	0.82 ± 0.12	0.85 ± 0.06	0.85 ± 0.06	0.84 ± 0.09	0.69 ± 0.12	
ExtraTreesClassifier	0.84 ± 0.04	0.86 ± 0.08	0.82 ± 0.12	0.84 ± 0.04	0.84 ± 0.04	0.84 ± 0.09	0.67 ± 0.08	
GradientBoostingClassifier	0.83 ± 0.06	0.83 ± 0.06	0.83 ± 0.12	0.83 ± 0.06	0.83 ± 0.05	0.84 ± 0.1	0.66 ± 0.11	
QuantumNearestMeanCentroid	0.85 ± 0.08	0.9 ± 0.06	0.8 ± 0.15	0.85 ± 0.08	0.84 ± 0.12	0.86 ± 0.07	0.7 ± 0.16	
HelstromQuantumCentroid1	0.84 ± 0.07	0.84 ± 0.1	0.85 ± 0.11	0.84 ± 0.08	0.86 ± 0.08	0.84 ± 0.07	0.68 ± 0.15	0.28 ± 0.01
HelstromQuantumCentroid2	0.88 ± 0.06	0.91 ± 0.07	0.85 ± 0.07	0.88 ± 0.06	0.86 ± 0.07	0.89 ± 0.06	0.76 ± 0.11	0.22 ± 0.01
HelstromQuantumCentroid3	0.89 ± 0.06	0.92 ± 0.07	0.86 ± 0.07	0.89 ± 0.06	0.87 ± 0.07	0.89 ± 0.05	0.77 ± 0.12	0.2 ± 0.0
HelstromQuantumCentroid4	0.88 ± 0.06	0.9 ± 0.07	0.87 ± 0.07	0.89 ± 0.06	0.87 ± 0.09	0.88 ± 0.07	0.77 ± 0.13	0.18 ± 0.0

Table 2. Prnn_synth dataset (with $\#n$ number of copies)

Classifier	Accuracy	Sensitivity	Specificity	Balanced Accuracy	Precision	F-measure	Cohen's k	Helstrom Error
BernoulliNB	0.56 ± 0.06	0.48 ± 0.1	0.64 ± 0.03	0.56 ± 0.06	0.52 ± 0.09	0.57 ± 0.07	0.12 ± 0.12	
LogisticRegression	0.52 ± 0.06	0.4 ± 0.02	0.64 ± 0.11	0.52 ± 0.06	0.46 ± 0.04	0.54 ± 0.09	0.04 ± 0.12	
GaussianNB	0.5 ± 0.04	0.32 ± 0.01	0.68 ± 0.07	0.5 ± 0.04	0.39 ± 0.03	0.51 ± 0.06	0.0 ± 0.08	
KNeighborsClassifier	0.54 ± 0.0	0.4 ± 0.02	0.68 ± 0.01	0.54 ± 0.0	0.47 ± 0.01	0.56 ± 0.0	0.08 ± 0.0	
RandomForestClassifier	0.46 ± 0.0	0.56 ± 0.06	0.36 ± 0.05	0.46 ± 0.0	0.51 ± 0.03	0.47 ± 0.0	-0.08 ± 0.0	
AdaBoostClassifier	0.52 ± 0.02	0.4 ± 0.02	0.64 ± 0.03	0.52 ± 0.02	0.46 ± 0.02	0.53 ± 0.03	0.04 ± 0.04	
NearestCentroid	0.6 ± 0.02	0.4 ± 0.02	0.8 ± 0.05	0.6 ± 0.02	0.5 ± 0.0	0.67 ± 0.04	0.2 ± 0.03	
LinearDiscriminantAnalysis	0.52 ± 0.06	0.36 ± 0.03	0.69 ± 0.15	0.52 ± 0.06	0.43 ± 0.01	0.56 ± 0.11	0.04 ± 0.12	
QuadraticDiscriminantAnalysis	0.56 ± 0.02	0.36 ± 0.03	0.76 ± 0.07	0.56 ± 0.02	0.45 ± 0.01	0.61 ± 0.06	0.12 ± 0.04	
ExtraTreesClassifier	0.44 ± 0.02	0.44 ± 0.06	0.44 ± 0.02	0.44 ± 0.02	0.44 ± 0.04	0.44 ± 0.02	-0.12 ± 0.04	
GradientBoostingClassifier	0.46 ± 0.04	0.32 ± 0.01	0.6 ± 0.1	0.46 ± 0.04	0.37 ± 0.01	0.45 ± 0.05	-0.08 ± 0.08	
QuantumNearestMeanCentroid	0.5 ± 0.02	0.52 ± 0.06	0.48 ± 0.02	0.5 ± 0.02	0.5 ± 0.0	0.51 ± 0.03	0.0 ± 0.04	
HelstromQuantumCentroid1	0.68 ± 0.0	0.46 ± 0.35	0.72 ± 0.28	0.59 ± 0.04	0.86 ± 0.14	0.48 ± 0.28	0.2 ± 0.07	0.38 ± 0.02
HelstromQuantumCentroid2	0.44 ± 0.0	0.44 ± 0.1	0.44 ± 0.1	0.44 ± 0.0	0.43 ± 0.03	0.43 ± 0.07	-0.13 ± 0.0	0.34 ± 0.01
HelstromQuantumCentroid3	0.52 ± 0.04	0.53 ± 0.1	0.52 ± 0.02	0.53 ± 0.04	0.52 ± 0.02	0.52 ± 0.04	0.05 ± 0.08	0.22 ± 0.0
HelstromQuantumCentroid4	0.6 ± 0.04	0.57 ± 0.07	0.66 ± 0.16	0.61 ± 0.05	0.64 ± 0.14	0.58 ± 0.02	0.22 ± 0.09	0.24 ± 0.03

Table 3. Analcatdata_aids dataset (with #n number of copies)

Classifier	Accuracy	Sensitivity	Specificity	Balanced Accuracy	Precision	F-measure	Cohen's k	Helstrom Error
BernoulliNB	0.74 ± 0.0	1.0 ± 0.0	0.0 ± 0.0	0.5 ± 0.0	0.85 ± 0.0	0.74 ± 0.0	0.0 ± 0.0	
LogisticRegression	0.74 ± 0.01	0.95 ± 0.03	0.15 ± 0.05	0.55 ± 0.01	0.84 ± 0.01	0.76 ± 0.01	0.13 ± 0.02	
GaussianNB	0.74 ± 0.01	0.94 ± 0.02	0.16 ± 0.01	0.55 ± 0.0	0.84 ± 0.01	0.76 ± 0.0	0.13 ± 0.01	
KNeighborsClassifier	0.7 ± 0.03	0.88 ± 0.01	0.21 ± 0.14	0.54 ± 0.06	0.81 ± 0.01	0.76 ± 0.03	0.09 ± 0.14	
RandomForestClassifier	0.7 ± 0.02	0.89 ± 0.04	0.17 ± 0.05	0.53 ± 0.01	0.81 ± 0.01	0.75 ± 0.0	0.07 ± 0.01	
AdaBoostClassifier	0.73 ± 0.02	0.88 ± 0.01	0.3 ± 0.1	0.59 ± 0.04	0.83 ± 0.01	0.78 ± 0.02	0.2 ± 0.09	
NearestCentroid	0.71 ± 0.01	0.79 ± 0.03	0.48 ± 0.03	0.63 ± 0.0	0.8 ± 0.01	0.81 ± 0.0	0.26 ± 0.0	
LinearDiscriminantAnalysis	0.74 ± 0.01	0.95 ± 0.03	0.16 ± 0.04	0.56 ± 0.0	0.84 ± 0.01	0.76 ± 0.0	0.14 ± 0.0	
QuadraticDiscriminantAnalysis	0.75 ± 0.01	0.92 ± 0.01	0.26 ± 0.09	0.59 ± 0.04	0.84 ± 0.01	0.78 ± 0.02	0.22 ± 0.08	
ExtraTreesClassifier	0.71 ± 0.01	0.89 ± 0.04	0.2 ± 0.08	0.55 ± 0.02	0.82 ± 0.01	0.76 ± 0.01	0.11 ± 0.04	
GradientBoostingClassifier	0.7 ± 0.01	0.84 ± 0.04	0.33 ± 0.07	0.58 ± 0.01	0.8 ± 0.01	0.78 ± 0.01	0.18 ± 0.02	
QuantumNearestMeanCentroid	0.61 ± 0.07	0.65 ± 0.13	0.51 ± 0.12	0.58 ± 0.01	0.79 ± 0.01	0.71 ± 0.08	0.14 ± 0.03	
HelstromQuantumCentroid1	0.63 ± 0.06	0.61 ± 0.1	0.69 ± 0.02	0.65 ± 0.04	0.84 ± 0.03	0.7 ± 0.08	0.24 ± 0.06	0.42 ± 0.01
HelstromQuantumCentroid2	0.66 ± 0.02	0.71 ± 0.03	0.53 ± 0.01	0.62 ± 0.02	0.81 ± 0.02	0.76 ± 0.02	0.22 ± 0.03	0.36 ± 0.01
HelstromQuantumCentroid3	0.69 ± 0.01	0.76 ± 0.0	0.48 ± 0.04	0.62 ± 0.02	0.8 ± 0.05	0.78 ± 0.03	0.22 ± 0.05	0.31 ± 0.01
HelstromQuantumCentroid4	0.57 ± 0.0	0.6 ± 0.03	0.46 ± 0.07	0.53 ± 0.02	0.76 ± 0.0	0.67 ± 0.02	0.05 ± 0.04	0.27 ± 0.01

Table 4. Haberman dataset (with #n number of copies)

Classifier	Accuracy	Sensitivity	Specificity	Balanced Accuracy	Precision	F-measure	Cohen's k	Helstrom Error
BernoulliNB	0.82 ± 0.05	0.66 ± 0.12	0.99 ± 0.01	0.82 ± 0.05	0.78 ± 0.08	0.99 ± 0.01	0.65 ± 0.11	
LogisticRegression	0.86 ± 0.02	0.85 ± 0.09	0.87 ± 0.05	0.86 ± 0.02	0.86 ± 0.03	0.87 ± 0.03	0.72 ± 0.04	
GaussianNB	0.88 ± 0.02	0.88 ± 0.08	0.88 ± 0.04	0.88 ± 0.02	0.88 ± 0.03	0.88 ± 0.03	0.76 ± 0.04	
KNeighborsClassifier	1.0 ± 0.0	1.0 ± 0.0	1.0 ± 0.0	1.0 ± 0.0	1.0 ± 0.0	1.0 ± 0.0	1.0 ± 0.0	
RandomForestClassifier	0.96 ± 0.0	0.97 ± 0.03	0.95 ± 0.03	0.96 ± 0.0	0.96 ± 0.0	0.95 ± 0.03	0.92 ± 0.0	
AdaBoostClassifier	0.97 ± 0.03	0.97 ± 0.03	0.97 ± 0.03	0.97 ± 0.03	0.97 ± 0.03	0.97 ± 0.03	0.94 ± 0.06	
NearestCentroid	0.78 ± 0.03	0.79 ± 0.11	0.78 ± 0.06	0.78 ± 0.03	0.78 ± 0.04	0.79 ± 0.02	0.57 ± 0.05	
LinearDiscriminantAnalysis	0.88 ± 0.02	0.88 ± 0.08	0.88 ± 0.04	0.88 ± 0.02	0.88 ± 0.03	0.88 ± 0.03	0.76 ± 0.04	
QuadraticDiscriminantAnalysis	0.88 ± 0.03	0.87 ± 0.09	0.88 ± 0.04	0.88 ± 0.02	0.87 ± 0.03	0.88 ± 0.02	0.75 ± 0.05	
ExtraTreesClassifier	1.0 ± 0.0	1.0 ± 0.0	1.0 ± 0.0	1.0 ± 0.0	1.0 ± 0.0	1.0 ± 0.0	1.0 ± 0.0	
GradientBoostingClassifier	0.96 ± 0.02	0.97 ± 0.03	0.95 ± 0.01	0.96 ± 0.02	0.96 ± 0.02	0.95 ± 0.01	0.92 ± 0.04	
QuantumNearestMeanCentroid	0.89 ± 0.0	0.88 ± 0.1	0.9 ± 0.1	0.89 ± 0.0	0.92 ± 0.08	0.89 ± 0.01	0.78 ± 0.0	
HelstromQuantumCentroid1	0.86 ± 0.04	0.74 ± 0.04	0.99 ± 0.01	0.87 ± 0.03	0.99 ± 0.01	0.85 ± 0.03	0.73 ± 0.07	0.24 ± 0.01
HelstromQuantumCentroid2	0.94 ± 0.0	0.97 ± 0.01	0.9 ± 0.01	0.94 ± 0.0	0.9 ± 0.02	0.94 ± 0.01	0.87 ± 0.01	0.2 ± 0.01
HelstromQuantumCentroid3	0.98 ± 0.02	1.0 ± 0.0	0.96 ± 0.04	0.98 ± 0.02	0.96 ± 0.04	0.98 ± 0.02	0.96 ± 0.04	0.09 ± 0.01
HelstromQuantumCentroid4	1.0 ± 0.0	1.0 ± 0.0	1.0 ± 0.0	1.0 ± 0.0	1.0 ± 0.0	1.0 ± 0.0	1.0 ± 0.0	0.06 ± 0.0

Table 5. Moon dataset (with #n number of copies)

Classifier	Accuracy	Sensitivity	Specificity	Balanced Accuracy	Precision	F-measure	Cohen's k	Helstrom Error
BernoulliNB	0.6 ± 0.01	0.0 ± 0.0	1.0 ± 0.0	0.5 ± 0.0	0.0 ± 0.0	0.0 ± 0.0	0.0 ± 0.0	
LogisticRegression	0.7 ± 0.07	0.58 ± 0.13	0.79 ± 0.02	0.68 ± 0.07	0.6 ± 0.1	0.64 ± 0.07	0.37 ± 0.15	
GaussianNB	0.67 ± 0.08	0.55 ± 0.27	0.75 ± 0.06	0.65 ± 0.11	0.54 ± 0.18	0.57 ± 0.07	0.29 ± 0.2	
KNeighborsClassifier	0.72 ± 0.05	0.54 ± 0.01	0.85 ± 0.08	0.69 ± 0.04	0.61 ± 0.05	0.72 ± 0.12	0.4 ± 0.1	
RandomForestClassifier	0.68 ± 0.0	0.66 ± 0.05	0.69 ± 0.04	0.68 ± 0.0	0.62 ± 0.01	0.59 ± 0.02	0.34 ± 0.0	
AdaBoostClassifier	0.7 ± 0.0	0.57 ± 0.07	0.79 ± 0.06	0.68 ± 0.01	0.6 ± 0.02	0.65 ± 0.04	0.37 ± 0.01	
NearestCentroid	0.72 ± 0.03	0.75 ± 0.14	0.71 ± 0.13	0.73 ± 0.0	0.68 ± 0.02	0.66 ± 0.08	0.45 ± 0.02	
LinearDiscriminantAnalysis	0.75 ± 0.03	0.66 ± 0.1	0.81 ± 0.12	0.73 ± 0.01	0.68 ± 0.01	0.73 ± 0.11	0.47 ± 0.03	
QuadraticDiscriminantAnalysis	0.64 ± 0.1	0.47 ± 0.3	0.77 ± 0.04	0.62 ± 0.13	0.47 ± 0.24	0.51 ± 0.14	0.23 ± 0.25	
ExtraTreesClassifier	0.66 ± 0.0	0.66 ± 0.1	0.65 ± 0.08	0.66 ± 0.01	0.6 ± 0.03	0.56 ± 0.02	0.3 ± 0.02	
GradientBoostingClassifier	0.71 ± 0.04	0.54 ± 0.04	0.83 ± 0.1	0.69 ± 0.03	0.6 ± 0.02	0.7 ± 0.11	0.38 ± 0.07	
QuantumNearestMeanCentroid	0.72 ± 0.02	0.4 ± 0.01	0.94 ± 0.06	0.67 ± 0.02	0.85 ± 0.15	0.54 ± 0.02	0.38 ± 0.05	
HelstromQuantumCentroid1	0.69 ± 0.03	0.23 ± 0.17	0.98 ± 0.02	0.61 ± 0.09	0.75 ± 0.25	0.34 ± 0.23	0.23 ± 0.19	0.42 ± 0.03
HelstromQuantumCentroid2	0.7 ± 0.12	0.32 ± 0.22	0.98 ± 0.02	0.65 ± 0.1	0.94 ± 0.06	0.42 ± 0.24	0.33 ± 0.22	0.38 ± 0.04
HelstromQuantumCentroid3	0.71 ± 0.06	0.32 ± 0.09	0.98 ± 0.02	0.65 ± 0.06	0.9 ± 0.1	0.47 ± 0.12	0.33 ± 0.13	0.37 ± 0.05
HelstromQuantumCentroid4	0.71 ± 0.01	0.3 ± 0.1	0.98 ± 0.02	0.64 ± 0.06	0.88 ± 0.12	0.44 ± 0.13	0.31 ± 0.11	0.37 ± 0.03

Table 6. Lupus dataset (with $\#n$ number of copies)

Classifier	Accuracy	Sensitivity	Specificity	Balanced Accuracy	Precision	F-measure	Cohen's k	Helstrom Error
BernoulliNB	0.52 ± 0.01	0.34 ± 0.3	0.7 ± 0.3	0.52 ± 0.01	0.33 ± 0.24	0.76 ± 0.24	0.04 ± 0.01	
LogisticRegression	0.53 ± 0.01	0.53 ± 0.01	0.54 ± 0.03	0.53 ± 0.01	0.53 ± 0.0	0.53 ± 0.01	0.06 ± 0.01	
GaussianNB	0.77 ± 0.02	0.66 ± 0.07	0.88 ± 0.03	0.77 ± 0.02	0.74 ± 0.04	0.84 ± 0.01	0.54 ± 0.05	
KNeighborsClassifier	0.72 ± 0.04	0.61 ± 0.03	0.84 ± 0.04	0.72 ± 0.04	0.69 ± 0.04	0.79 ± 0.05	0.44 ± 0.08	
RandomForestClassifier	0.69 ± 0.01	0.68 ± 0.01	0.7 ± 0.02	0.69 ± 0.01	0.69 ± 0.02	0.7 ± 0.02	0.38 ± 0.03	
AdaBoostClassifier	0.7 ± 0.04	0.65 ± 0.07	0.76 ± 0.02	0.7 ± 0.04	0.68 ± 0.05	0.72 ± 0.03	0.4 ± 0.09	
NearestCentroid	0.52 ± 0.04	0.5 ± 0.03	0.55 ± 0.05	0.52 ± 0.04	0.51 ± 0.03	0.53 ± 0.04	0.05 ± 0.08	
LinearDiscriminantAnalysis	0.54 ± 0.01	0.52 ± 0.01	0.56 ± 0.03	0.54 ± 0.01	0.53 ± 0.0	0.54 ± 0.01	0.09 ± 0.02	
QuadraticDiscriminantAnalysis	0.78 ± 0.02	0.66 ± 0.06	0.89 ± 0.02	0.77 ± 0.02	0.74 ± 0.03	0.86 ± 0.01	0.55 ± 0.04	
ExtraTreesClassifier	0.68 ± 0.02	0.69 ± 0.04	0.68 ± 0.0	0.68 ± 0.02	0.69 ± 0.03	0.68 ± 0.01	0.37 ± 0.04	
GradientBoostingClassifier	0.7 ± 0.02	0.66 ± 0.03	0.74 ± 0.01	0.7 ± 0.02	0.69 ± 0.03	0.72 ± 0.01	0.4 ± 0.04	
QuantumNearestMeanCentroid	0.74 ± 0.01	0.66 ± 0.02	0.81 ± 0.03	0.74 ± 0.01	0.78 ± 0.02	0.72 ± 0.0	0.47 ± 0.01	
HelstromQuantumCentroid1	0.67 ± 0.03	0.46 ± 0.13	0.89 ± 0.08	0.68 ± 0.02	0.84 ± 0.1	0.57 ± 0.09	0.35 ± 0.06	0.41 ± 0.01
HelstromQuantumCentroid2	0.76 ± 0.02	0.6 ± 0.0	0.91 ± 0.03	0.76 ± 0.02	0.87 ± 0.04	0.71 ± 0.01	0.51 ± 0.03	0.31 ± 0.01
HelstromQuantumCentroid3	0.76 ± 0.03	0.62 ± 0.03	0.89 ± 0.02	0.76 ± 0.03	0.86 ± 0.02	0.72 ± 0.03	0.52 ± 0.06	0.31 ± 0.01
HelstromQuantumCentroid4	0.76 ± 0.02	0.63 ± 0.06	0.88 ± 0.08	0.76 ± 0.01	0.86 ± 0.07	0.72 ± 0.01	0.52 ± 0.03	0.29 ± 0.0

Table 7. GaussianPlos dataset (with $\#n$ number of copies)

Classifier	Accuracy	Sensitivity	Specificity	Balanced Accuracy	Precision	F-measure	Cohen's k	Helstrom Error
BernoulliNB	0.77 ± 0.01	0.46 ± 0.0	0.92 ± 0.02	0.69 ± 0.01	0.57 ± 0.01	0.74 ± 0.05	0.43 ± 0.02	
LogisticRegression	0.78 ± 0.02	0.49 ± 0.02	0.92 ± 0.01	0.7 ± 0.02	0.59 ± 0.03	0.73 ± 0.04	0.44 ± 0.04	
GaussianNB	0.77 ± 0.01	0.52 ± 0.0	0.89 ± 0.01	0.71 ± 0.01	0.6 ± 0.01	0.7 ± 0.02	0.45 ± 0.02	
KNeighborsClassifier	0.78 ± 0.0	0.44 ± 0.06	0.95 ± 0.03	0.69 ± 0.01	0.57 ± 0.03	0.81 ± 0.07	0.44 ± 0.02	
RandomForestClassifier	0.78 ± 0.01	0.44 ± 0.06	0.95 ± 0.04	0.69 ± 0.01	0.56 ± 0.02	0.83 ± 0.12	0.44 ± 0.01	
AdaBoostClassifier	0.78 ± 0.01	0.44 ± 0.06	0.95 ± 0.04	0.69 ± 0.01	0.56 ± 0.02	0.83 ± 0.12	0.44 ± 0.01	
NearestCentroid	0.75 ± 0.01	0.59 ± 0.01	0.82 ± 0.0	0.71 ± 0.01	0.6 ± 0.01	0.62 ± 0.01	0.42 ± 0.01	
LinearDiscriminantAnalysis	0.78 ± 0.02	0.49 ± 0.02	0.92 ± 0.01	0.7 ± 0.02	0.59 ± 0.03	0.73 ± 0.04	0.44 ± 0.04	
QuadraticDiscriminantAnalysis	0.77 ± 0.01	0.52 ± 0.0	0.89 ± 0.01	0.71 ± 0.01	0.6 ± 0.01	0.7 ± 0.02	0.45 ± 0.02	
ExtraTreesClassifier	0.78 ± 0.01	0.44 ± 0.06	0.95 ± 0.04	0.69 ± 0.01	0.56 ± 0.02	0.83 ± 0.12	0.44 ± 0.01	
GradientBoostingClassifier	0.78 ± 0.01	0.44 ± 0.06	0.95 ± 0.04	0.69 ± 0.01	0.56 ± 0.02	0.83 ± 0.12	0.44 ± 0.01	
QuantumNearestMeanCentroid	0.68 ± 0.08	0.66 ± 0.04	0.68 ± 0.13	0.67 ± 0.04	0.52 ± 0.08	0.57 ± 0.03	0.32 ± 0.1	
HelstromQuantumCentroid1	0.75 ± 0.0	0.6 ± 0.01	0.81 ± 0.0	0.71 ± 0.0	0.61 ± 0.0	0.61 ± 0.0	0.42 ± 0.0	0.35 ± 0.0
HelstromQuantumCentroid2	0.75 ± 0.01	0.6 ± 0.03	0.81 ± 0.01	0.71 ± 0.01	0.61 ± 0.0	0.61 ± 0.02	0.42 ± 0.02	0.3 ± 0.01
HelstromQuantumCentroid3	0.76 ± 0.03	0.56 ± 0.03	0.85 ± 0.06	0.71 ± 0.01	0.66 ± 0.07	0.6 ± 0.01	0.43 ± 0.04	0.34 ± 0.01
HelstromQuantumCentroid4	0.76 ± 0.02	0.56 ± 0.03	0.85 ± 0.04	0.71 ± 0.0	0.65 ± 0.05	0.6 ± 0.0	0.43 ± 0.02	0.33 ± 0.0

Table 8. Titanic dataset (with $\#n$ number of copies)

Classifier	Accuracy	Sensitivity	Specificity	Balanced Accuracy	Precision	F-measure	Cohen's k	Helstrom Error
BernoulliNB	0.63 ± 0.02	0.9 ± 0.1	0.14 ± 0.14	0.52 ± 0.02	0.76 ± 0.03	0.66 ± 0.01	0.04 ± 0.04	
LogisticRegression	0.63 ± 0.0	0.9 ± 0.03	0.14 ± 0.05	0.52 ± 0.01	0.76 ± 0.01	0.66 ± 0.01	0.05 ± 0.02	
GaussianNB	0.69 ± 0.06	0.87 ± 0.05	0.36 ± 0.07	0.61 ± 0.06	0.79 ± 0.04	0.72 ± 0.03	0.25 ± 0.14	
KNeighborsClassifier	0.66 ± 0.06	0.78 ± 0.09	0.43 ± 0.0	0.61 ± 0.04	0.75 ± 0.05	0.72 ± 0.02	0.22 ± 0.1	
RandomForestClassifier	0.66 ± 0.01	0.78 ± 0.01	0.43 ± 0.0	0.61 ± 0.01	0.75 ± 0.01	0.72 ± 0.0	0.22 ± 0.01	
AdaBoostClassifier	0.77 ± 0.02	0.85 ± 0.05	0.62 ± 0.05	0.73 ± 0.0	0.82 ± 0.02	0.81 ± 0.01	0.48 ± 0.02	
NearestCentroid	0.54 ± 0.01	0.54 ± 0.0	0.55 ± 0.02	0.54 ± 0.01	0.6 ± 0.0	0.69 ± 0.01	0.08 ± 0.02	
LinearDiscriminantAnalysis	0.63 ± 0.0	0.82 ± 0.03	0.29 ± 0.05	0.55 ± 0.01	0.74 ± 0.01	0.68 ± 0.01	0.12 ± 0.02	
QuadraticDiscriminantAnalysis	0.69 ± 0.04	0.88 ± 0.01	0.33 ± 0.1	0.61 ± 0.05	0.79 ± 0.02	0.71 ± 0.03	0.24 ± 0.12	
ExtraTreesClassifier	0.65 ± 0.03	0.73 ± 0.04	0.5 ± 0.02	0.62 ± 0.03	0.73 ± 0.03	0.73 ± 0.02	0.23 ± 0.06	
GradientBoostingClassifier	0.79 ± 0.02	0.86 ± 0.01	0.67 ± 0.05	0.76 ± 0.03	0.84 ± 0.02	0.83 ± 0.02	0.53 ± 0.06	
QuantumNearestMeanCentroid	0.54 ± 0.02	0.46 ± 0.02	0.68 ± 0.07	0.57 ± 0.03	0.73 ± 0.01	0.57 ± 0.01	0.12 ± 0.05	
HelstromQuantumCentroid1	0.48 ± 0.0	0.27 ± 0.05	0.88 ± 0.04	0.57 ± 0.01	0.81 ± 0.01	0.4 ± 0.06	0.11 ± 0.01	0.45 ± 0.01
HelstromQuantumCentroid2	0.54 ± 0.02	0.43 ± 0.07	0.74 ± 0.04	0.59 ± 0.01	0.75 ± 0.02	0.55 ± 0.06	0.14 ± 0.03	0.4 ± 0.0
HelstromQuantumCentroid3	0.48 ± 0.03	0.42 ± 0.09	0.6 ± 0.07	0.51 ± 0.01	0.66 ± 0.01	0.51 ± 0.07	0.02 ± 0.02	0.36 ± 0.03
HelstromQuantumCentroid4	0.54 ± 0.04	0.42 ± 0.01	0.76 ± 0.1	0.59 ± 0.05	0.77 ± 0.08	0.55 ± 0.03	0.15 ± 0.09	0.36 ± 0.02

Table 9. Analcatdata_boxing1 dataset (with #n number of copies)

Classifier	Accuracy	Sensitivity	Specificity	Balanced Accuracy	Precision	F-measure	Cohen's k	Helstrom Error
BernoulliNB	0.82 ± 0.04	0.89 ± 0.06	0.76 ± 0.02	0.83 ± 0.04	0.81 ± 0.04	0.75 ± 0.03	0.64 ± 0.08	
LogisticRegression	0.82 ± 0.04	0.84 ± 0.0	0.8 ± 0.07	0.82 ± 0.03	0.81 ± 0.04	0.78 ± 0.06	0.64 ± 0.07	
GaussianNB	0.81 ± 0.0	0.81 ± 0.03	0.8 ± 0.02	0.81 ± 0.01	0.79 ± 0.01	0.77 ± 0.01	0.61 ± 0.01	
KNeighborsClassifier	0.77 ± 0.06	0.76 ± 0.03	0.78 ± 0.09	0.77 ± 0.06	0.75 ± 0.06	0.74 ± 0.09	0.54 ± 0.12	
RandomForestClassifier	0.84 ± 0.01	0.76 ± 0.03	0.91 ± 0.0	0.83 ± 0.02	0.81 ± 0.02	0.87 ± 0.01	0.68 ± 0.03	
AdaBoostClassifier	0.8 ± 0.03	0.73 ± 0.01	0.85 ± 0.07	0.79 ± 0.03	0.76 ± 0.03	0.8 ± 0.06	0.58 ± 0.06	
NearestCentroid	0.81 ± 0.05	0.89 ± 0.06	0.74 ± 0.04	0.81 ± 0.05	0.8 ± 0.05	0.73 ± 0.05	0.62 ± 0.1	
LinearDiscriminantAnalysis	0.82 ± 0.06	0.89 ± 0.0	0.76 ± 0.11	0.83 ± 0.06	0.82 ± 0.05	0.76 ± 0.09	0.64 ± 0.12	
QuadraticDiscriminantAnalysis	0.81 ± 0.0	0.81 ± 0.03	0.8 ± 0.02	0.81 ± 0.01	0.79 ± 0.01	0.77 ± 0.01	0.61 ± 0.01	
ExtraTreesClassifier	0.86 ± 0.03	0.87 ± 0.02	0.85 ± 0.07	0.86 ± 0.02	0.84 ± 0.02	0.83 ± 0.06	0.71 ± 0.05	
GradientBoostingClassifier	0.8 ± 0.03	0.78 ± 0.01	0.8 ± 0.07	0.79 ± 0.03	0.78 ± 0.02	0.77 ± 0.05	0.59 ± 0.06	
QuantumNearestMeanCentroid	0.81 ± 0.03	0.84 ± 0.05	0.78 ± 0.01	0.81 ± 0.03	0.76 ± 0.01	0.79 ± 0.03	0.61 ± 0.05	
HelstromQuantumCentroid1	0.8 ± 0.01	0.81 ± 0.03	0.78 ± 0.05	0.8 ± 0.01	0.75 ± 0.03	0.78 ± 0.0	0.59 ± 0.02	0.29 ± 0.0
HelstromQuantumCentroid2	0.78 ± 0.07	0.71 ± 0.19	0.82 ± 0.02	0.77 ± 0.09	0.76 ± 0.06	0.73 ± 0.13	0.55 ± 0.16	0.19 ± 0.04
HelstromQuantumCentroid3	0.8 ± 0.03	0.82 ± 0.05	0.8 ± 0.1	0.81 ± 0.02	0.76 ± 0.14	0.78 ± 0.05	0.59 ± 0.07	0.18 ± 0.02
HelstromQuantumCentroid4	0.78 ± 0.05	0.75 ± 0.21	0.74 ± 0.09	0.74 ± 0.06	0.69 ± 0.1	0.71 ± 0.15	0.5 ± 0.13	0.16 ± 0.04

Table 10. Analcatdata_asbestos dataset (with #n number of copies)

Classifier	Accuracy	Sensitivity	Specificity	Balanced Accuracy	Precision	F-measure	Cohen's k	Helstrom Error
BernoulliNB	0.8 ± 0.01	0.15 ± 0.15	0.96 ± 0.04	0.56 ± 0.06	0.19 ± 0.19	0.25 ± 0.25	0.13 ± 0.13	
LogisticRegression	0.87 ± 0.04	0.47 ± 0.17	0.96 ± 0.01	0.72 ± 0.09	0.57 ± 0.17	0.74 ± 0.14	0.5 ± 0.19	
GaussianNB	0.88 ± 0.03	0.52 ± 0.12	0.96 ± 0.01	0.74 ± 0.07	0.62 ± 0.12	0.77 ± 0.1	0.55 ± 0.13	
KNeighborsClassifier	0.86 ± 0.01	0.52 ± 0.12	0.94 ± 0.01	0.73 ± 0.05	0.58 ± 0.08	0.68 ± 0.02	0.5 ± 0.09	
RandomForestClassifier	0.88 ± 0.03	0.52 ± 0.12	0.96 ± 0.01	0.74 ± 0.07	0.62 ± 0.12	0.77 ± 0.1	0.55 ± 0.13	
AdaBoostClassifier	0.88 ± 0.01	0.48 ± 0.02	0.98 ± 0.0	0.73 ± 0.01	0.61 ± 0.02	0.83 ± 0.0	0.54 ± 0.02	
NearestCentroid	0.84 ± 0.01	0.71 ± 0.01	0.87 ± 0.01	0.79 ± 0.01	0.64 ± 0.03	0.58 ± 0.04	0.54 ± 0.04	
LinearDiscriminantAnalysis	0.87 ± 0.04	0.47 ± 0.17	0.96 ± 0.01	0.72 ± 0.09	0.57 ± 0.17	0.74 ± 0.14	0.5 ± 0.19	
QuadraticDiscriminantAnalysis	0.86 ± 0.05	0.47 ± 0.17	0.95 ± 0.02	0.71 ± 0.1	0.56 ± 0.18	0.69 ± 0.19	0.48 ± 0.21	
ExtraTreesClassifier	0.85 ± 0.0	0.34 ± 0.06	0.98 ± 0.02	0.66 ± 0.02	0.46 ± 0.04	0.83 ± 0.17	0.39 ± 0.02	
GradientBoostingClassifier	0.85 ± 0.03	0.53 ± 0.07	0.93 ± 0.02	0.73 ± 0.05	0.58 ± 0.08	0.65 ± 0.1	0.49 ± 0.11	
QuantumNearestMeanCentroid	0.81 ± 0.02	0.82 ± 0.07	0.81 ± 0.04	0.82 ± 0.01	0.52 ± 0.08	0.63 ± 0.04	0.51 ± 0.04	
HelstromQuantumCentroid1	0.85 ± 0.02	0.64 ± 0.07	0.89 ± 0.02	0.77 ± 0.03	0.58 ± 0.08	0.61 ± 0.08	0.51 ± 0.06	0.25 ± 0.01
HelstromQuantumCentroid2	0.79 ± 0.02	0.76 ± 0.06	0.8 ± 0.04	0.78 ± 0.01	0.49 ± 0.01	0.59 ± 0.01	0.46 ± 0.0	0.17 ± 0.02
HelstromQuantumCentroid3	0.84 ± 0.01	0.71 ± 0.11	0.87 ± 0.01	0.79 ± 0.05	0.57 ± 0.03	0.63 ± 0.06	0.53 ± 0.06	0.14 ± 0.03
HelstromQuantumCentroid4	0.89 ± 0.04	0.81 ± 0.03	0.91 ± 0.04	0.86 ± 0.04	0.69 ± 0.15	0.73 ± 0.1	0.66 ± 0.12	0.11 ± 0.01

Table 11. Appendicitis dataset (with #n number of copies)

Classifier	Accuracy	Sensitivity	Specificity	Balanced Accuracy	Precision	F-measure	Cohen's k	Helstrom Error
BernoulliNB	0.53 ± 0.01	0.87 ± 0.13	0.13 ± 0.13	0.5 ± 0.0	0.66 ± 0.04	0.54 ± 0.0	0.0 ± 0.0	
LogisticRegression	0.65 ± 0.01	0.7 ± 0.01	0.59 ± 0.01	0.65 ± 0.01	0.69 ± 0.01	0.67 ± 0.01	0.3 ± 0.02	
GaussianNB	0.7 ± 0.03	0.77 ± 0.14	0.61 ± 0.09	0.69 ± 0.03	0.73 ± 0.06	0.7 ± 0.01	0.39 ± 0.06	
KNeighborsClassifier	0.6 ± 0.03	0.66 ± 0.12	0.53 ± 0.07	0.59 ± 0.02	0.63 ± 0.06	0.62 ± 0.0	0.19 ± 0.05	
RandomForestClassifier	0.62 ± 0.01	0.69 ± 0.0	0.54 ± 0.03	0.62 ± 0.01	0.66 ± 0.0	0.64 ± 0.01	0.23 ± 0.02	
AdaBoostClassifier	0.74 ± 0.03	0.7 ± 0.04	0.77 ± 0.03	0.74 ± 0.03	0.74 ± 0.04	0.78 ± 0.03	0.47 ± 0.07	
NearestCentroid	0.63 ± 0.0	0.65 ± 0.02	0.61 ± 0.03	0.63 ± 0.0	0.65 ± 0.01	0.66 ± 0.01	0.25 ± 0.01	
LinearDiscriminantAnalysis	0.66 ± 0.03	0.72 ± 0.06	0.59 ± 0.01	0.65 ± 0.03	0.69 ± 0.04	0.67 ± 0.01	0.31 ± 0.05	
QuadraticDiscriminantAnalysis	0.64 ± 0.05	0.72 ± 0.14	0.54 ± 0.06	0.63 ± 0.04	0.67 ± 0.07	0.64 ± 0.02	0.26 ± 0.09	
ExtraTreesClassifier	0.64 ± 0.01	0.66 ± 0.12	0.62 ± 0.11	0.64 ± 0.0	0.66 ± 0.05	0.68 ± 0.03	0.28 ± 0.01	
GradientBoostingClassifier	0.7 ± 0.04	0.68 ± 0.01	0.72 ± 0.08	0.7 ± 0.04	0.71 ± 0.03	0.74 ± 0.06	0.4 ± 0.08	
QuantumNearestMeanCentroid	0.63 ± 0.02	0.66 ± 0.09	0.61 ± 0.05	0.63 ± 0.02	0.66 ± 0.05	0.65 ± 0.02	0.26 ± 0.04	
HelstromQuantumCentroid1	0.62 ± 0.03	0.64 ± 0.06	0.61 ± 0.0	0.62 ± 0.03	0.65 ± 0.01	0.64 ± 0.02	0.24 ± 0.06	0.4 ± 0.0
HelstromQuantumCentroid2	0.61 ± 0.08	0.72 ± 0.16	0.51 ± 0.02	0.61 ± 0.09	0.62 ± 0.01	0.66 ± 0.08	0.22 ± 0.18	0.35 ± 0.04
HelstromQuantumCentroid3	0.64 ± 0.02	0.69 ± 0.19	0.6 ± 0.18	0.65 ± 0.0	0.68 ± 0.08	0.66 ± 0.06	0.29 ± 0.02	0.34 ± 0.02
HelstromQuantumCentroid4	0.61 ± 0.05	0.62 ± 0.02	0.62 ± 0.09	0.62 ± 0.06	0.65 ± 0.11	0.63 ± 0.06	0.23 ± 0.11	0.31 ± 0.03

Table 12. Analcatdata_boxing2 dataset (with $\#n$ number of copies)

Classifier	Accuracy	Sensitivity	Specificity	Balanced Accuracy	Precision	F-measure	Cohen's k	Helstrom Error
BernoulliNB	0.5 ± 0.0	0.62 ± 0.38	0.38 ± 0.38	0.5 ± 0.0	0.49 ± 0.17	0.5 ± 0.0	0.0 ± 0.0	
LogisticRegression	0.51 ± 0.01	0.23 ± 0.02	0.79 ± 0.03	0.51 ± 0.01	0.32 ± 0.01	0.52 ± 0.02	0.02 ± 0.01	
GaussianNB	0.52 ± 0.01	0.17 ± 0.03	0.86 ± 0.05	0.52 ± 0.01	0.26 ± 0.03	0.57 ± 0.06	0.03 ± 0.03	
KNeighborsClassifier	0.51 ± 0.01	0.51 ± 0.05	0.51 ± 0.03	0.51 ± 0.01	0.51 ± 0.03	0.51 ± 0.01	0.01 ± 0.02	
RandomForestClassifier	0.48 ± 0.01	0.5 ± 0.02	0.46 ± 0.0	0.48 ± 0.01	0.49 ± 0.02	0.48 ± 0.01	-0.04 ± 0.03	
AdaBoostClassifier	0.5 ± 0.0	0.4 ± 0.0	0.61 ± 0.0	0.5 ± 0.0	0.44 ± 0.0	0.5 ± 0.0	0.01 ± 0.01	
NearestCentroid	0.51 ± 0.01	0.23 ± 0.02	0.79 ± 0.03	0.51 ± 0.01	0.32 ± 0.01	0.52 ± 0.02	0.02 ± 0.01	
LinearDiscriminantAnalysis	0.51 ± 0.01	0.23 ± 0.02	0.79 ± 0.03	0.51 ± 0.01	0.32 ± 0.01	0.52 ± 0.02	0.02 ± 0.01	
QuadraticDiscriminantAnalysis	0.52 ± 0.01	0.17 ± 0.03	0.86 ± 0.05	0.52 ± 0.01	0.26 ± 0.03	0.57 ± 0.06	0.03 ± 0.03	
ExtraTreesClassifier	0.48 ± 0.01	0.48 ± 0.01	0.48 ± 0.0	0.48 ± 0.01	0.48 ± 0.01	0.48 ± 0.01	-0.04 ± 0.01	
GradientBoostingClassifier	0.51 ± 0.01	0.53 ± 0.04	0.48 ± 0.02	0.51 ± 0.01	0.52 ± 0.02	0.51 ± 0.01	0.01 ± 0.02	
QuantumNearestMeanCentroid	0.5 ± 0.01	0.51 ± 0.27	0.49 ± 0.29	0.5 ± 0.01	0.51 ± 0.01	0.47 ± 0.14	-0.0 ± 0.02	
HelstromQuantumCentroid1	0.5 ± 0.01	0.5 ± 0.4	0.5 ± 0.38	0.5 ± 0.01	0.48 ± 0.03	0.4 ± 0.24	-0.01 ± 0.01	0.5 ± 0.0
HelstromQuantumCentroid2	0.52 ± 0.0	0.17 ± 0.01	0.87 ± 0.03	0.52 ± 0.02	0.58 ± 0.09	0.26 ± 0.02	0.04 ± 0.04	0.48 ± 0.01
HelstromQuantumCentroid3	0.54 ± 0.01	0.31 ± 0.0	0.78 ± 0.04	0.54 ± 0.02	0.58 ± 0.05	0.4 ± 0.01	0.08 ± 0.03	0.48 ± 0.01
HelstromQuantumCentroid4	0.5 ± 0.01	0.28 ± 0.19	0.72 ± 0.17	0.5 ± 0.01	0.49 ± 0.03	0.32 ± 0.17	0.0 ± 0.02	0.47 ± 0.0

Table 13. Hill_Valley_with_noise dataset (with $\#n$ number of copies)

Classifier	Accuracy	Sensitivity	Specificity	Balanced Accuracy	Precision	F-measure	Cohen's k	Helstrom Error
BernoulliNB	0.5 ± 0.0	0.91 ± 0.09	0.09 ± 0.09	0.5 ± 0.0	0.65 ± 0.02	0.5 ± 0.0	-0.0 ± 0.0	
LogisticRegression	0.52 ± 0.01	0.87 ± 0.01	0.16 ± 0.02	0.51 ± 0.01	0.64 ± 0.0	0.51 ± 0.0	0.03 ± 0.01	
GaussianNB	0.51 ± 0.0	0.88 ± 0.0	0.13 ± 0.01	0.51 ± 0.0	0.65 ± 0.0	0.51 ± 0.0	0.02 ± 0.01	
KNeighborsClassifier	0.5 ± 0.03	0.5 ± 0.05	0.5 ± 0.0	0.5 ± 0.03	0.5 ± 0.04	0.5 ± 0.03	-0.01 ± 0.05	
RandomForestClassifier	0.49 ± 0.01	0.53 ± 0.0	0.45 ± 0.02	0.49 ± 0.01	0.51 ± 0.0	0.49 ± 0.01	-0.02 ± 0.02	
AdaBoostClassifier	0.49 ± 0.01	0.49 ± 0.06	0.49 ± 0.04	0.49 ± 0.01	0.49 ± 0.04	0.5 ± 0.01	-0.01 ± 0.02	
NearestCentroid	0.51 ± 0.01	0.82 ± 0.01	0.2 ± 0.03	0.51 ± 0.01	0.63 ± 0.0	0.51 ± 0.01	0.02 ± 0.02	
LinearDiscriminantAnalysis	0.52 ± 0.01	0.87 ± 0.01	0.16 ± 0.02	0.52 ± 0.01	0.65 ± 0.0	0.51 ± 0.0	0.03 ± 0.02	
QuadraticDiscriminantAnalysis	0.51 ± 0.0	0.88 ± 0.0	0.13 ± 0.01	0.51 ± 0.0	0.65 ± 0.0	0.51 ± 0.0	0.02 ± 0.01	
ExtraTreesClassifier	0.51 ± 0.01	0.53 ± 0.01	0.48 ± 0.01	0.51 ± 0.01	0.52 ± 0.01	0.51 ± 0.01	0.01 ± 0.01	
GradientBoostingClassifier	0.51 ± 0.01	0.52 ± 0.0	0.5 ± 0.02	0.51 ± 0.01	0.52 ± 0.0	0.52 ± 0.01	0.03 ± 0.01	
QuantumNearestMeanCentroid	0.53 ± 0.0	0.78 ± 0.03	0.26 ± 0.03	0.52 ± 0.0	0.52 ± 0.0	0.63 ± 0.01	0.05 ± 0.0	
HelstromQuantumCentroid1	0.52 ± 0.01	0.81 ± 0.01	0.23 ± 0.0	0.52 ± 0.0	0.52 ± 0.02	0.63 ± 0.02	0.04 ± 0.01	0.48 ± 0.0
HelstromQuantumCentroid2	0.53 ± 0.01	0.77 ± 0.02	0.27 ± 0.03	0.52 ± 0.01	0.52 ± 0.02	0.62 ± 0.02	0.04 ± 0.01	0.47 ± 0.01
HelstromQuantumCentroid3	0.53 ± 0.0	0.82 ± 0.0	0.24 ± 0.0	0.53 ± 0.0	0.52 ± 0.01	0.64 ± 0.01	0.06 ± 0.01	0.48 ± 0.0
HelstromQuantumCentroid4	0.53 ± 0.01	0.81 ± 0.01	0.24 ± 0.02	0.53 ± 0.0	0.52 ± 0.02	0.64 ± 0.01	0.05 ± 0.01	0.48 ± 0.0

Table 14. Hill_Valley_without_noise dataset (with $\#n$ number of copies)

Position	Classifier	Average Success Rate
#1	HelstromQuantumCentroid4	72.8%
#2	HelstromQuantumCentroid3	65.13%
#3	GaussianNB	58%
#4	HelstromQuantumCentroid2	57.07%
#5	HelstromQuantumCentroid1	56.6%
#5	QuadraticDiscriminantAnalysis	56.6%
#6	GradientBoostingClassifier	52.73%
#7	ExtreTreesClassifier	51.93%
#8	KNeighborsClassifier	51.47%
#9	NearestCentroid	49.13%
#10	RandomForestClassifier	45.87%
#11	QuantumNearestMeanCentroid	43.93%
#12	AdaBoostClassifier	42.93%
#13	LinearDiscriminantAnalysis	42%
#14	LogisticRegression	36.4%
#15	BernoulliNB	17.4%

Table 15. Average Success Rate
